# Clinical and laboratory parameters associated with acute kidney injury in patients with snakebite envenomation: a prospective observational study from Myanmar

**DOI:** 10.1186/s12882-017-0510-0

**Published:** 2017-03-16

**Authors:** Kyi-Phyu Aye, Vipa Thanachartwet, Chit Soe, Varunee Desakorn, Khin-Thida Thwin, Supat Chamnanchanunt, Duangjai Sahassananda, Thanom Supaporn, Visith Sitprija

**Affiliations:** 1 Department of Clinical Tropical Medicine, Faculty of Tropical Medicine, Mahidol University, Bangkok, 10400 Thailand; 2 Medical Ward (I), 1000 Bedded Hospital, Naypyitaw, 15011 Myanmar; 3 Department of Rheumatology, University of Medicine 1, Lanmadaw, Yangon, 11131 Myanmar; 4 Department of Nephrology, University of Medicine 1, Lanmadaw, Yangon, 11131 Myanmar; 5 Information Technology Unit, Faculty of Tropical Medicine, Mahidol University, Bangkok, 10400 Thailand; 6 Division of Nephrology, Phramongkutklao Hospital, Bangkok, 10400 Thailand; 7 Division of Nephrology, Department of Medicine, Faculty of Medicine, Chulalongkorn University and King Chulalongkorn Memorial Hospital, Bangkok, 10330 Thailand; 8 Queen Saovabha Memorial Institute, Thai Red Cross, Bangkok, 10330 Thailand

**Keywords:** Clinical factors, Laboratory factors, Myanmar, Multivariate analysis, Snakebite-related acute kidney injury, Prospective study

## Abstract

**Background:**

Snakebite-related acute kidney injury (AKI) is a common community-acquired AKI in tropical countries leading to death and disability. The aims of this study were to (1) determine the occurrence of snakebite-related AKI, (2) assess factors at presentation that are associated with snakebite-related AKI, and (3) determine the outcomes of patients with snakebite-related AKI.

**Methods:**

We conducted a prospective observational study of patients with snake envenomation at the three academic tertiary care hospitals in Yangon, Myanmar between March 2015 and June 2016. Patient data including baseline characteristics, clinical and laboratory findings, hospital management, and outcomes were recorded in a case report form. A stepwise multivariate logistic regression analysis using a backward selection method determined independent factors significantly associated with AKI.

**Results:**

AKI was observed in 140 patients (54.3%), the majority of whom were AKI stage III (110 patients, 78.6%). AKI occurred at presentation and developed during hospitalization in 88 (62.9%) and 52 patients (37.1%), respectively. Twenty-seven patients died (19.3%), and 69 patients (49.3%) required dialysis. On multivariate logistic regression analysis, (1) snakebites from the *Viperidae* family (odds ratio [OR]: 9.65, 95% confidence interval [CI]: 2.42–38.44; *p* = 0.001), (2) WBC >10 × 10^3^ cells/μL (OR: 3.55, 95% CI: 1.35–9.34; *p* = 0.010), (3) overt disseminated intravascular coagulation (OR: 2.23, 95% CI: 1.02–4.89; *p* = 0.045), (4) serum creatine kinase >500 IU/L (OR: 4.06, 95% CI: 1.71–9.63; *p* = 0.001), (5) serum sodium <135 mmol/L (OR: 4.37, 95% CI: 2.04–9.38; *p* < 0.001), (6) presence of microscopic hematuria (OR: 3.60, 95% CI: 1.45–8.91; *p* = 0.006), and (7) duration from snakebite to receiving antivenom ≥2 h (OR: 3.73, 95% CI: 1.48–9.37; *p* = 0.005) were independently associated with AKI. Patients bitten by *Viperidae* with normal renal function who had serum sodium <135 mmol/L had a significantly higher urine sodium-to-creatinine ratio than those with serum sodium ≥135 mmol/L (*p* < 0.001).

**Conclusions:**

Identifying factors associated with snakebite-related AKI might help clinicians to be aware of snakebite patients who are at risk of AKI, particularly patients who demonstrate renal tubular dysfunction after *Viperidae* bites.

**Electronic supplementary material:**

The online version of this article (doi:10.1186/s12882-017-0510-0) contains supplementary material, which is available to authorized users.

## Background

Community-acquired acute kidney injury (cAKI) is a major public health problem in tropical countries, particularly in Asia [1, 2]. cAKI in tropical countries commonly affects young adults (age range 37–47 years) without pre-existing comorbidities [2]. These patients are at risk of developing chronic kidney disease [3]. cAKI in tropical countries is usually caused by a single etiology including tropical infections, environmental exposure to a toxin, or occupational risk of snakebite envenomation [1, 2]. South Asia, Southeast Asia, and sub-Saharan Africa have the highest burden of snakebite envenomation [4]. In Southeast Asia, envenomation by two families of venomous snakes, *Elapidae* and *Viperidae*, are associated with significant morbidity and mortality, with fatality rates of 0.4–20.0% [5, 6]. Following snakebite envenomation by snakes of the family *Viperidae* and *Colubridae*, patients may develop renal manifestations including proteinuria, hematuria, pigmenturia, and AKI [7, 8].

Snakebite-related AKI (sAKI) is a type of cAKI reported to affect from 8.0–43.0% of patients with snakebite envenomation [9–15], among whom approximately 15.0–55.0% required renal replacement therapy (RRT) [9–11, 13] and the fatality rate was 8.0–39.0% [9–11, 13, 14]. Previous reports from Brazil have shown greater susceptibility to sAKI with increasing age [16, 17]. Reported factors associated with sAKI included age <12 years, time from hospitalization to antivenom treatment >2 h, time from snakebite to receiving antivenom >2 h, longer duration from snakebite to hospital arrival, cellulitis, regional lymphadenopathy, hypotension, higher total bilirubin level, lower hemoglobin level, intravascular hemolysis, incoagulable blood on 20-min whole blood clotting test (20WBCT), prolonged bleeding time, prolonged prothrombin time (PT), hemorrhagic manifestations, serum creatine kinase >2000 IU/L, dark or brown urine color, albuminuria, and longer length of hospitalization [9, 10, 12, 13, 15].

A recent report from Myanmar found that approximately 10,000 cases of snakebite occurred annually. Russell’s viper was the most common source of envenomation, accounting for 90% of snakebite cases with the case fatality rate of 10.4% [18]. Another study from Myanmar showed that approximately 42% of patients with Russell’s viper bites (10 of 24 patients) developed sAKI, all of whom recovered after antivenom treatment, while another 21% of the snakebite patients (5 of 24) developed sAKI following antivenom treatment [19]. However, data on the renal manifestations and factors associated with the development of sAKI in Myanmar are limited. Overall, the factors associated with sAKI varied across studies due to differences in the study population, potency and composition of snake venom, which differs across geographic regions of the study sites; accessibility of management facilities; and study design [9, 10, 12, 13, 15–17].

Therefore, a prospective observational study was conducted in the three academic tertiary care hospitals in Yangon, Myanmar between March 2015 and June 2016 among patients with snakebite envenomation with the aims to assess (1) the occurrence of sAKI from presentation until discharge, (2) clinical and laboratory factors at presentation that independently associated with the development of sAKI, and (3) outcomes of patients with sAKI. This information might help clinicians to identify patients who are at risk for sAKI in order to provide optimal management for decreasing the incidence of cAKI in tropical countries.

## Methods

### Study design and population

This prospective observational study was conducted at the three academic tertiary care hospitals in Yangon, Myanmar. These hospitals treated the majority of snakebite victims in Yangon and had facilities for laboratory investigations as well as patient care. The procedure indicated by the Standards for the Reporting of Observation Studies in Epidemiology (STROBE) was followed [20]. The study’s inclusion criteria were patients at least 12 years old and presenting with clinical parameters of snake envenomation. Patients with (1) a history of underlying medical illness including diabetes mellitus, hypertension, neurological diseases, cardiovascular diseases, renal diseases, pulmonary diseases, liver diseases, and hematologic diseases; (2) receiving any antiplatelet or anticoagulant drugs; or (3) currently pregnant were excluded from this study. All snakebite patients in this study were admitted to the hospitals for observation and management. All patients received standard care by the treating physicians according to the Myanmar National Guidelines, which follow the World Health Organization (WHO) 2010 guidelines for the management of snakebites in Southeast Asian countries [6], with the slight modification of adding 20WBCT monitoring every 2 h for 12 h, and then 4 h for 12 h. Neurological symptoms and signs were observed every 30 min for 12 h, and then hourly for 12 h in cases of unknown snakebite.

Laboratory parameters including complete blood counts, incoagulable blood on 20WBCT, PT, international normalized ratio (INR), activated partial thromboplastin time (aPTT), fibrinogen level, D-dimer, blood glucose, electrolytes, serum urea, serum creatinine, liver function test, and electrocardiography (ECG) were evaluated at presentation and then as appropriate. Chest X-ray was performed when abnormal lung sounds were detected or upon clinical suspicion of respiratory complications. Urinalysis and spot urine analysis for sodium, potassium, protein, and creatinine were also performed at presentation. Spot urine tests for calcium and phosphate were performed when patients demonstrated hypocalcemia and hypophosphatemia, respectively. Spot urine test for magnesium was performed in patients with hypokalemia. Abnormal urine findings and electrolyte abnormalities are defined in Additional file 1: Table S1.

Treatment outcomes including duration of hospitalization, requirement of RRT, and survival status were summarized on discharge. Patient data including baseline characteristics, location where the snakebite occurred, anatomical site of the bite, type of snake, pre-hospital management, clinical parameters, laboratory findings, hospital management, and outcomes were recorded in a pre-defined case report form.

### Clinical parameters of envenoming from snakes

The clinical parameters of both local and systemic envenoming were defined according to the WHO 2010 guidelines for the management of snakebites (Additional file 1: Table S2) [6]. Local bleeding was defined as prolonged bleeding from fang marks, prolonged bleeding from venipuncture site, and/or bleeding from a recent wound. Spontaneous systemic bleeding manifested as skin bleeding (defined as petechiae, purpura, and/or ecchymosis), mucosal bleeding (defined as gum bleeding, hematemesis, melena, hematochezia, gross hematuria, and/or vaginal bleeding), and/or conjunctival bleeding. Hypotension was defined as systolic blood pressure of <90 mmHg without evidence of tissue hypoperfusion. Capillary leakage was defined as a rise in hematocrit >2.0% above the reference range of adults adjusted for sex and serum albumin <3.0 g/dL. Disseminated intravascular coagulation (DIC) was diagnosed according to the International Society for Thrombosis and Haemostasis (ISTH) scoring system for DIC, with a score ≥5 considered indicative of overt DIC. The score was calculated based on platelet (PLT) count (≥100 × 10^3^/μL = 0, <100 × 10^3^/μL = 1, <50 × 10^3^/μL = 2); D-dimer (<0.5 μg/L = 0, 0.5 to <1.0 μg/L = 1, ≥1.0 to <2.0 μg/L = 2, ≥2 μg/L = 3); PT (<15 s = 0, ≥15 s = 1, >20 s = 2); and fibrinogen level (≥100 mg/dL = 0, <100 mg/dL = 1) [21].

### Management of patients with snake envenoming

Antivenom treatment was indicated when patients developed local envenoming with the clinical signs of (1) local swelling in more than half of the bitten limb (in the absence of a tourniquet) within 48 h of the bite, (2) rapid extension of swelling over 1 joint within 2 h of a bite on the hands or feet, and/or (3) development of tender lymphadenitis. Antivenom was also indicated for all cases of systemic envenoming, as indicated by the following: (1) hemostatic abnormalities including spontaneous systemic bleeding, incoagulable blood on 20WBCT, and/or thrombocytopenia defined as PLT count <100 × 10^3^/μL; (2) neurotoxic signs including ptosis, external opthalmoplegia, and/or muscle paralysis; (3) cardiovascular abnormalities including hypotension, shock, and/or abnormal ECG; and/or (4) renal abnormalities including reduced urine volume and/or AKI.

Reduced urine volume was classified as oliguria, defined as <400 mL of urine volume in 24 h, or anuria, defined as no urine volume in 24 h. AKI was defined as the increase of serum creatinine ≥0.3 mg/dL within 48 h or increase in serum creatinine ≥1.5 times baseline. AKI staging was performed according to the Kidney Disease: Improving Global Outcomes (KDIGO) clinical practice guidelines. AKI stage I was defined as serum creatinine increased to 1.5 to 1.9 times baseline. AKI stage II was defined as serum creatinine increased to 2.0 to 2.9 times baseline. AKI stage III was defined as serum creatinine increased to 3.0 times baseline or higher, serum creatinine ≥4.0 mg/dL, or receipt of RRT [22]. The dosage of acute peritoneal dialysis was assessed using a weekly Kt/V measurement of urea according to the International Society of Peritoneal Dialysis (ISPD) guideline [23]. ‘K’ was defined as the volume of dialysate drained multiplied by dialysate/plasma urea concentration, and ‘t’ was defined as the duration of dialysis over 1 week. ‘V’ was defined as the volume of distribution of urea (total body water = 0.5 [female] or 0.6 [male] multiplied by body weight).

A specific antivenom treatment was prescribed based on snake identification by well-trained investigators using a handbook of the dangerous venomous snakes of Myanmar [24]. Snake identification was performed if the snake that definitely bit the patient was brought to the hospital either dead or alive for identification or based on the patients’ description of the snake to the investigators. If the snake could not be identified, the clinical syndrome of envenoming was considered according to the WHO 2010 guidelines for specific antivenom treatment [6]. The clinical syndrome of *Viperidae* envenoming consisted of presentation with (1) local envenoming and (2) clinical bleeding and/or incoagulable blood on 20WBCT. The clinical syndrome of Russell’s viper envenoming consisted of presentation with (1) local envenoming, (2) clinical bleeding and/or incoagulable blood on 20WBCT, and (3) shock defined by a systolic blood pressure of <90 mmHg with evidence of tissue hypoperfusion, i.e., <0.5 mL/kg/h decrease in urine output, cold skin, and/or receipt of inotropic drugs. The clinical syndrome of cobra envenoming consisted of presentation with (1) local envenoming and (2) muscle paralysis.

### Sample size calculation

We estimated the required sample size based on a 2013 study from India, which reported a 15% rate of AKI occurrence among patients with snake envenoming [14]. Based on this data, a minimum of 196 venomous snakebite patients were needed to achieve this rate with 5% margin of error. We predicted a dropout rate of 20% during this study; therefore, the required sample size was at least 235 patients.

### Statistical analyses

All data were analyzed using SPSS software (version 18.0; SPSS Inc., Chicago, IL). Numerical variables were tested for normality using the Kolmogorov-Smirnov test. Variables with non-normal distribution were summarized as medians and interquartile ranges (IQRs) and compared using Mann-Whitney *U* tests for two-group comparisons and Kruskal-Wallis tests for greater than two-group comparisons. A significant result on Kruskal-Wallis tests was subjected to further post-hoc pair-wise comparison by using the Mann-Whitney *U* test with Bonferroni correction to adjust significance values. Categorical variables were expressed as frequencies and percentages and analyzed using chi-squared or Fisher’s exact tests, as appropriate. A univariate logistic regression analysis was used to determine which of the collected baseline characteristics, pre-hospital management, clinical parameters, laboratory findings, and management, were associated with AKI among patients with venomous snakebites. All clinical factors potentially associated with AKI were included in the univariate logistic regression analysis as independent variables, with the occurrence of AKI as the dependent variable. Any variable with a *p* value ≤ 0.2 was included in a stepwise multivariate logistic regression analysis using a backward selection method for determining significant independent factors. Linear regression analysis was used to predict urine sodium-to-creatinine ratio from serum sodium and urine potassium-to-creatinine ratio from serum potassium. All tests of significance were two-sided, with a *p* value ≤ 0.05 indicating statistical significance.

## Results

**Fig. 1:**
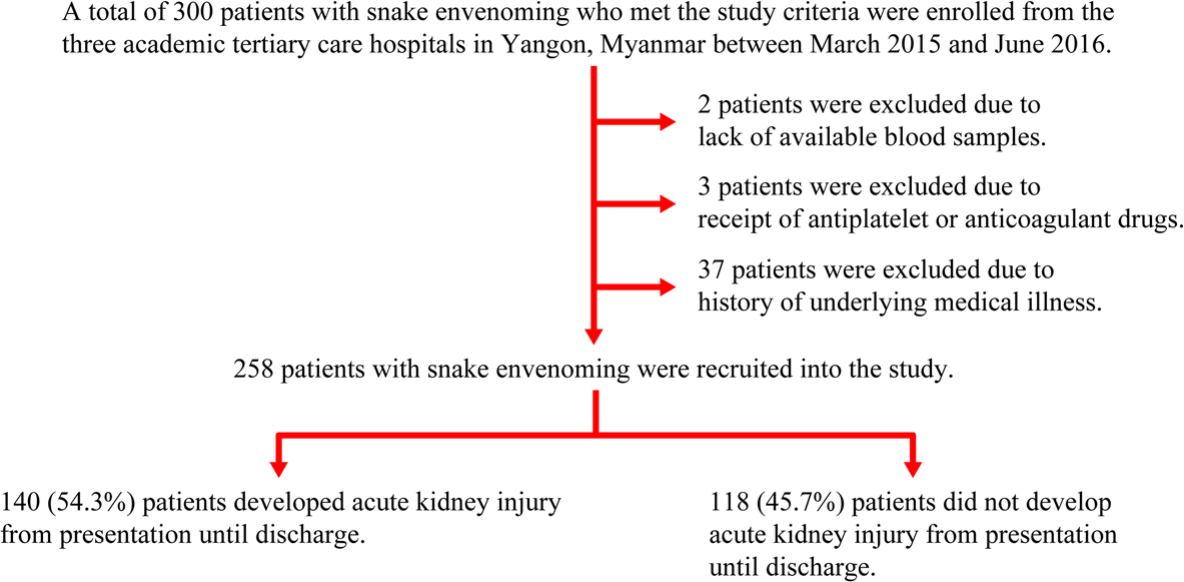
Flow diagram showing the recruitment of study patients

A total of 300 patients with snake envenoming were enrolled from the three academic tertiary care hospitals in Yangon, Myanmar between March 2015 and June 2016. Of 300 patients with venomous snakebites, 42 patients were excluded due to a lack of available blood samples (2 patients), receipt of antiplatelet or anticoagulant drugs (3 patients), and history of underlying medical illness (37 patients). Thus, 258 patients with venomous snakebites were ultimately recruited for this study (Fig. 1). Snake identification was possible in 174 patients (67.4%). Identified snakes included Russell’s viper (147 patients, 84.5%), cobra (20 patients, 11.5%), green pit viper (6 patients, 3.4%), and sea snake (1 patient, 0.6%). The median (IQR) age of patients with venomous snakebites was 31.0 (23.0–42.0) years, and the majority of patients were male (203 patients, 78.7%). Renal manifestations among patients with venomous snakebites at presentation are shown in Table 1. Of 128 patients with reduced urine volume, 112 (87.5%) patients presented with oliguria, and 16 (12.5%) patients presented with anuria. Of 140 patients with AKI, 88 (62.9%) had AKI at presentation, and 52 (37.1%) developed AKI during hospitalization. Among the 52 patients who developed AKI during hospitalization, 48 (92.3%) developed AKI within 48 h of hospitalization, and the remaining 4 (7.7%) developed AKI after 48 h of hospitalization. AKI stages I and II were observed in 15 (10.7%) patients each, and AKI stage III was observed in 110 patients (78.6%). Thus, the 258 patients with venomous snakebites included 140 (54.3%) patients with AKI and 118 (45.7%) patients without AKI (Fig. 1).

**Table 1:** Renal manifestations among 258 adults with snakebite envenomation, Yangon, Myanmar, 2015–2016

Characteristic	All	*n* (%)
*Clinical manifestations*		
Reduced urine volume	258	128 (49.6)
Renal tenderness	258	114 (44.2)
Gross hematuria	258	46 (17.8)
Dark- colored urine	258	25 (9.7)
*Urinalysis and urine chemistries*		
Urine protein-to-creatinine ratio ≥1	186	112 (60.2)
Microscopic hematuria	255	81 (31.4)
Pigmenturia	255	38 (14.7)
Leukocyturia	255	22 (8.5)
*Blood chemistry*		
Acute kidney injury	258	140 (54.3)
At presentation	140	88 (62.9)
During hospitalization	140	52 (37.1)
Serum sodium		
Hyponatremia	258	115 (44.6)
Hypernatremia	258	5 (1.9)
Serum potassium		
Hypokalemia	258	52 (20.2)
Hyperkalemia	258	19 (7.4)
Serum calcium		
Hypocalcemia	256	88 (34.4)
Hypercalcemia	256	6 (2.3)
Serum phosphate		
Hypophosphatemia	256	20 (7.8)
Hyperphosphatemia	256	68 (26.6)
Serum bicarbonate		
Metabolic acidosis	258	22 (8.5)
Metabolic alkalosis	258	1 (0.4)

### Comparison of baseline characteristics, pre-hospital management, clinical, and laboratory parameters between patients with and without AKI

Baseline characteristics and pre-hospital management are shown in Additional file 1: Table S3. Most baseline characteristics were similar, except that the rates of patients living in Yangon city or being bitten in the field, on the lower extremities, by a snake of the *Viperidae* family, or presenting with the clinical syndrome of *Viperidae* envenoming (all *p* < 0.001) were more likely to develop AKI. Most pre-hospital management characteristics were also similar between the groups, except that tourniquet application (*p* = 0.015) and receipt of management by a traditional healer (*p* = 0.048) were associated with the development of AKI.

Clinical presentations that were associated with the development of AKI included tender lymphadenitis, local swelling grades II to IV, bruising, vomiting, muscle pain, abdominal pain, abnormal ECG, hypotension, shock, conjunctival edema, pulmonary edema, systemic bleeding, reduced urine volume, renal tenderness, puffy eyelids (all *p* < 0.001), and dark-colored urine (*p* = 0.003) (Additional file 1: Table S4). Laboratory findings at presentation are shown in Additional file 1: Table S5. Patients with AKI had significantly higher WBC counts and neutrophil levels (both *p* < 0.001), PT (*p* = 0.036), INR (*p* = 0.019), aPTT (*p* = 0.005), fibrinogen (*p* = 0.013), D-dimer (*p* < 0.001), blood sugar (*p* = 0.002), serum urea, serum creatinine, serum potassium, serum phosphate, serum creatine kinase, AST, ALT, urine protein-to-creatinine ratio, and fractional excretion of sodium (all *p* < 0.001). In addition, there were significantly higher numbers of patients with incoagulable blood on 20WBCT and microscopic hematuria (all *p* < 0.001) among those who developed AKI. However, patients with AKI had significantly lower lymphocyte level, PLT count, serum sodium, serum chloride, serum bicarbonate, serum calcium, albumin, and fractional excretion of urea (all *p* = 0.001). When laboratory findings were categorized based on reference ranges, a significantly higher proportion of patients with WBC count >10 × 10^3^ cells/μL (*p* < 0.001), overt DIC (*p* < 0.001), presence of capillary leakage (*p* < 0.001), serum creatine kinase >500 IU/L (*p* < 0.001), blood sugar ≥150 mg/dL (*p* = 0.017), and serum sodium <135 mmol/L (*p* < 0.001) developed AKI. Potential factors related to the development of AKI including clinical and laboratory parameters are summarized in Table 2. For any potential factors that had multi-collinearity with each other, only one appropriate factor was chosen.

**Table 2:** Clinical parameters, laboratory findings, and management characteristics among 258 adults with snakebite envenomation, Yangon, Myanmar, 2015–2016

Characteristics	All	With acute kidney injury, *n* (%)	Without acute kidney injury, *n* (%)	*p* value
*Clinical parameters*				
Bites from *Viperidae* or presenting the clinical syndrome of *Viperidae*				
Yes	214	136 (63.6)	78 (36.4)	<0.001
No	44	4 (9.1)	40 (90.9)	
Local swelling				
Grades II–IV	167	111 (66.5)	56 (33.5)	<0.001
Grades 0-I	91	29 (31.9)	62 (68.1)	
Hypotension				
Yes	69	60 (87.0)	9 (13.0)	<0.001
No	189	80 (42.3)	109 (57.7)	
*Laboratory parameters*				
WBC (cells/μL)				
> 10 × 10^3^	194	126 (64.9)	68 (35.1)	<0.001
≤ 10 × 10^3^	64	14 (21.9)	50 (78.1)	
Overt DIC				
Yes	147	103 (70.1)	44 (29.9)	<0.001
No	111	37 (33.3)	74 (66.7)	
Capillary leakage				
Yes	35	30 (85.7)	5 (14.3)	<0.001
No	223	110 (49.3)	113 (50.7)	
Creatine kinase (IU/L)				
> 500	104	87 (83.7)	17 (16.3)	<0.001
≤ 500	152	51 (33.6)	101 (66.4)	
Blood sugar (mg/dL)				
≥ 150	53	37 (69.8)	16 (30.2)	0.017
< 150	205	103 (50.2)	102 (49.8)	
Serum sodium (mmol/L)				
< 135 mmol/L	115	91 (79.1)	24 (20.9)	<0.001
≥ 135 mmol/L	143	49 (34.3)	94 (65.7)	
Microscopic hematuria				
Yes	81	70 (86.4)	11 (13.6)	<0.001
No	174	67 (38.5)	107 (61.5)	
*Management*				
Site of management				
Ward	211	129 (61.1)	82 (38.9)	<0.001
ICU	47	11 (23.4)	36 (76.6)	
Time from bite to receiving antivenom (hours)				
≥ 2	174	107 (61.5)	67 (38.5)	0.007
< 2	76	32 (42.1)	44 (57.9)	

*Abbreviations*: *DIC* disseminated intravascular coagulation, *ICU* intensive care unit, *WBC* white blood cell, *20WBCT* 20-min whole blood clotting test

**Fig. 2:**
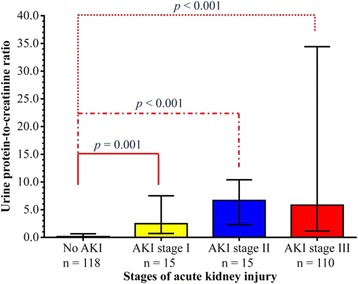
*Spot* urine protein-to-creatinine ratio at presentation among patients with different stages of acute kidney injury. Data are presented in *box* and *whisker plots* with median (*horizontal line*), interquartile range (*box*), and maximum values within 1.5 of interquartile range (*whiskers)*

Urine protein-to-creatinine ratio in relation to AKI staging or the absence of AKI was also evaluated. A significant different in median urine protein-to-creatinine ratio was observed among the groups (Kruskal-Wallis test, chi-squared = 66.572, degrees of freedom = 3, *p* < 0.001). Post-hoc analysis by Mann-Whitney *U* test with Bonferroni correction showed that the median (IQR) urine protein-to-creatinine ratio of patients without AKI (0.14 [0.03–0.65]) was significantly lower than those of patients with AKI stage I (2.47 [0.71–7.51], *p* = 0.001), AKI stage II (6.66 [2.24–10.39], *p* < 0.001), and AKI stage III (5.83 [1.15–34.41], *p* < 0.001). However, urine protein-to-creatinine ratio did not significantly differ among patients with AKI stage I, II, or III (Fig. 2).

### Comparison of management and outcomes among patients with and without AKI

The management and outcomes of patients with and without AKI are shown in Additional file 1: Table S6. A significantly higher proportion of patients with AKI had a duration from snakebite to hospital of ≥1 h (*p* = 0.037) and had a duration from snakebite to receipt of antivenom of ≥2 h (*p* = 0.007). Patients with AKI received a significantly higher total dose of antivenom treatment (*p* < 0.001). The proportion of patients who received monovalent or polyvalent antivenom treatment was similar in both groups. Significantly higher proportions of patients with AKI received antibiotics at admission, received inpatient treatment on a hospital ward, received inotropic drugs (all *p* < 0.001), and developed panhypopituitarism (*p* = 0.004). The potential management characteristics related to the development of AKI are shown in Table 2. Regarding patient outcomes, patients with AKI had a significantly longer duration of hospitalization, greater requirement for RRT, and higher mortality rate (all *p* < 0.001).

Of 140 patients with AKI, 27 (19.3%) died, and 113 (80.7%) survived. Clinical parameters and management of deceased and surviving patients with AKI are shown in Table 3. Patients who died were more likely to develop shock, receive inotropic drugs, and demonstrate acute respiratory distress syndrome (ARDS) or respiratory failure (all *p* < 0.001). Of 140 patients with AKI, 69 (49.3%) required RRT. The indications for dialysis included uremia in 39 patients (56.5%), severe metabolic acidosis in 15 (21.7%), fluid overload in 9 (13.0%), and severe hyperkalemia in 6 (8.7%). The proportions of patients who required RRT were similar between those who died and those who survived. Acute peritoneal dialysis was performed in 62 patients who required RRT (89.8%), and the remaining 7 patients (10.2%) received hemodialysis. The methods of dialysis used in both groups were similar (*p* = 0.118). Weekly Kt/V was evaluated in only 52 patients who received acute peritoneal dialysis, including 8 patients (15.4%) who died and 44 (84.6%) who survived. The median (IQR) weekly Kt/V was similar in both groups (1.9 [1.1–3.0] vs. 2.7 [2.2–3.9], *p* = 0.116).

**Table 3:** Clinical parameters and management among 140 adults with acute kidney injury (deceased and survivors)

Characteristics	Deceased		Survivors		*p* value
	All	*n* (%)	All	*n* (%)	
Time from bite to hospital ≥1 h	27	24 (88.9)	113	91 (80.5)	0.408
Time from bite to receiving antivenom ≥2 h	27	21 (77.8)	112	86 (76.8)	1.000
Management in ICU	27	7 (25.9)	113	4 (3.5)	0.001
Shock	27	20 (74.1)	113	34 (30.1)	<0.001
Received inotropic drugs	27	20 (74.1)	113	23 (20.4)	<0.001
ARDS or respiratory failure	27	13 (48.1)	113	9 (8.0)	<0.001
Required renal replacement therapy	27	13 (48.1)	113	56 (49.6)	1.000
Received peritoneal dialysis	13	10 (76.9)	56	52 (92.9)	0.118
Weekly Kt/v, median (IQR)	8	1.9 (1.1–3.0)	44	2.7 (2.2–3.9)	0.116

*Abbreviations*: *ARDS* acute respiratory distress syndrome, *ICU* intensive care unit, *IQR* interquartile range

### Univariate and multivariate analyses for identifying factors associated with AKI among patients with venomous snakebites

Using a univariate logistic regression model, the following clinical parameters were associated with AKI: snakebites of the family *Viperidae* or presenting the clinical syndrome of *Viperidae* envenoming, local swelling of grades II to IV, presence of hypotension, WBC count >10 × 10^3^ cells/μL, overt DIC, presence of capillary leakage, serum creatine kinase >500 IU/L, blood sugar ≥150 mg/dL, serum sodium <135 mmol/L, presence of microscopic hematuria, management on a ward, and duration from bite to receipt of antivenom ≥2 h (Table 4).

**Table 4:** Logistic regression analysis of parameters associated with acute kidney injury, Yangon, Myanmar, 2015–2016

Characteristic	Univariate analysis			Multivariate analysis		
	*n*	OR (95% CI)	*p* value	*n*	OR (95% CI)	*p* value
*Clinical parameters*						
Bites from *Viperidae* or presenting clinical syndrome of *Viperidae*	258			244		
Yes		17.44 (6.01–50.57)	<0.001		9.65 (2.42–38.44)	0.001
No		1.00 (Reference)			1.00 (Reference)	
Local swelling	258					
Grades II–IV		4.24 (2.45–7.31)	<0.001			
Grades 0-I		1.00 (Reference)				
Hypotension	258					
Yes		9.08 (4.26–19.38)	<0.001			
No		1.00 (Reference)				
*Laboratory parameters*						
WBC (cells/μL)	258			244		
> 10 × 10^3^		6.62 (3.41–12.83)	<0.001		3.55 (1.35–9.34)	0.010
≤ 10 × 10^3^		1.00 (Reference)			1.00 (Reference)	
Overt DIC	258			244		
Yes		4.68 (2.76–7.95)	<0.001		2.23 (1.02–4.89)	0.045
No		1.00 (Reference)			1.00 (Reference)	
Capillary leakage	258					
Yes		6.16 (2.31–16.46)	<0.001			
No		1.00 (Reference)				
Creatine kinase (IU/L)	256			244		
> 500		10.14 (5.46–18.83)	<0.001		4.06 (1.71–9.63)	0.001
≤ 500		1.00 (Reference)			1.00 (Reference)	
Blood sugar (mg/dL)	258					
≥ 150		2.29 (2.00–4.37)	0.012			
< 150		1.00 (Reference)				
Serum sodium (mmol/L)	258			244		
< 135 mmol/L		7.27 (4.13–12.82)	<0.001		4.37 (2.04–9.38)	<0.001
≥ 135 mmol/L		1.00 (Reference)			1.00 (Reference)	
Microscopic hematuria	255			244		
Yes		10.16 (5.02–20.57)	<0.001		3.60 (1.45–8.91)	0.006
No		1.00 (Reference)			1.00 (Reference)	
*Management*						
Site of management	258					
Ward		5.15 (2.48–10.68)	<0.001			
ICU		1.00 (Reference)				
Time from bite to receiving antivenom (hours)	250			244		
≥ 2		2.20 (1.27–3.80)	0.005		3.73 (1.48–9.37)	0.005
< 2		1.00 (Reference)			1.00 (Reference)	

*Abbreviations*: *CI* confidence interval, *DIC* disseminated intravascular coagulation, *ICU* intensive care unit, *OR* odds ratio, *WBC* white blood cell

In a multivariate logistic regression model, the following clinical and laboratory parameters were independently associated with AKI: snakebites of the family *Viperidae* or presenting the clinical syndrome of *Viperidae* envenoming (odds ratio [OR]: 9.65; 95% CI: 2.42–38.44; *p* = 0.001), WBC count >10 × 10^3^ cells/μL (OR: 3.55, 95% CI: 1.35–9.34; *p* = 0.010), overt DIC (OR: 2.23, 95% CI: 1.02–4.89; *p* = 0.045), serum creatine kinase >500 IU/L (OR: 4.06, 95% CI: 1.71–9.63; *p* = 0.001), serum sodium <135 mmol/L (OR: 4.37, 95% CI: 2.04–9.38; *p* < 0.001), presence of microscopic hematuria (OR: 3.60, 95% CI: 1.45–8.91; *p* = 0.006), and duration from snakebite to receipt of antivenom ≥2 h (OR: 3.73, 95% CI: 1.48–9.37; *p* = 0.005) (Table 4).

### Subgroup analysis for identifying factors associated with AKI at presentation and during hospitalization among patients bitten by *Viperidae* or presenting the clinical syndrome of *Viperidae*

AKI is a common complication after snakebite envenomation from members of the *Viperidae* family [5–8]. Thus, factors associated with AKI at presentation and during hospitalization among patients bitten by *Viperidae* or presenting the clinical syndrome of *Viperidae* envenoming were identified in this study. Of 214 patients bitten by *Viperidae* or presenting the clinical syndrome of *Viperidae* envenoming, 136 (63.6%) developed AKI, including 86 patients (63.2%) with AKI at presentation and 50 (36.8%) who developed AKI during hospitalization. The remaining 78 patients (36.4%) did not develop AKI. Of the 50 patients who developed AKI during hospitalization, 47 (94.0%) developed AKI within 48 h of hospitalization and the remaining 3 patients (6.0%) developed AKI after 48 h of hospitalization.

Comparisons of clinical parameters, laboratory findings, management, and outcomes between the 86 patients with AKI at presentation and 78 patients without AKI are shown in Additional file 1: Tables S7, S8, and S9. Clinical parameters, laboratory findings, and management characteristics possibly affecting renal function are shown in Table 5. Patients with the parameters of presence of hypotension (*p* < 0.001), WBC count >10 × 10^3^ cells/μL (*p* < 0.001), overt DIC (*p* < 0.001), presence of capillary leakage (*p* = 0.001), serum creatine kinase >500 IU/L (*p* < 0.001), blood sugar ≥150 mg/dL (*p* = 0.015), serum sodium <135 mmol/L (*p* < 0.001), presence of microscopic hematuria (*p* < 0.001), and duration from snakebite to receipt of antivenom ≥2 h (*p* = 0.021) were more likely to develop AKI at presentation (Table 5).

**Table 5:** Clinical parameters, laboratory findings, and management characteristics of 164 adults bitten by *Viperidae* or presenting the clinical syndrome of *Viperidae* (86 patients with acute kidney injury at presentation and 78 patients without acute kidney injury)

Characteristics	All	With acute kidney injury, *n* (%)	Without acute kidney injury, *n* (%)	*p* value
Hypotension				
Yes	46	39 (84.8)	7 (15.2)	<0.001
No	118	47 (39.8)	71 (60.2)	
WBC (cells/μL)				
> 10 × 10^3^	122	75 (61.5)	47 (38.5)	<0.001
≤ 10 × 10^3^	41	10 (24.4)	31 (75.6)	
Overt DIC				
Yes	100	64 (64.0)	36 (36.0)	<0.001
No	64	22 (34.4)	42 (65.6)	
Capillary leakage				
Yes	19	17 (89.5)	2 (10.5)	0.001
No	145	69 (47.6)	76 (52.4)	
Creatine kinase (IU/L)				
> 500	69	57 (82.6)	12 (17.4)	<0.001
≤ 500	95	29 (30.5)	66 (69.5)	
Blood sugar (mg/dL)				
≥ 150	38	27 (71.1)	11 (28.9)	0.015
< 150	126	59 (46.8)	67 (53.2)	
Serum sodium (mmol/L)				
< 135	77	61 (79.2)	16 (20.8)	<0.001
≥ 135	87	25 (28.7)	62 (71.3)	
Microscopic hematuria				
Yes	61	51 (83.6)	10 (16.4)	<0.001
No	101	33 (32.7)	68 (67.3)	
Time from bite to receiving antivenom (hours)				
≥ 2	112	68 (60.7)	44 (39.3)	0.021
< 2	44	17 (38.6)	27 (61.4)	

*Abbreviations*: *DIC* disseminated intravascular coagulation, *WBC* white blood cell

These parameters were also associated with AKI at presentation using a univariate logistic regression model (Table 6). In a multivariate logistic regression model, the following clinical and laboratory parameters were independently associated with AKI at presentation: presence of hypotension (odds ratio [OR]: 3.56; 95% CI: 1.05–12.09; *p* = 0.042), serum creatine kinase >500 IU/L (OR: 6.24, 95% CI: 2.10–18.49; *p* = 0.001), serum sodium <135 mmol/L (OR: 7.81, 95% CI: 2.87–21.24; *p* < 0.001), presence of microscopic hematuria (OR: 11.45, 95% CI: 3.76–34.82; *p* < 0.001), and duration from snakebite to receipt of antivenom ≥2 h (OR: 7.98, 95% CI: 2.06–30.92; *p* = 0.003) (Table 6).

**Table 6:** Univariate and multivariate analysis of clinical and laboratory parameters of adults bitten by *Viperidae* or presenting the clinical syndrome of *Viperidae* for identifying acute kidney injury at presentation

Characteristic	Univariate analysis			Multivariate analysis		
	*n*	OR (95% CI)	*p* value	*n*	OR (95% CI)	*p* value
Hypotension	164			153		
Yes		8.42 (3.47–20.39)	<0.001		3.56 (1.05–12.09)	0.042
No		1.00 (Reference)			1.00 (Reference)	
WBC (cells/μL)	163					
> 10 × 10^3^		4.95 (2.22–11.02)	<0.001			
≤ 10 × 10^3^		1.00 (Reference)				
Overt DIC	164					
Yes		3.39 (1.76–6.55)	<0.001			
No		1.00 (Reference)				
Capillary leakage	164					
Yes		9.36 (2.09–42.00)	0.003			
No		1.00 (Reference)				
Creatine kinase (IU/L)	164			153		
> 500		10.81 (5.05–23.12)	<0.001		6.24 (2.10–18.49)	0.001
≤ 500		1.00 (Reference)			1.00 (Reference)	
Blood sugar (mg/dL)	164					
≥ 150		2.79 (1.27–6.10)	0.010			
< 150		1.00 (Reference)				
Serum sodium (mmol/L)	164			153		
< 135 mmol/L		9.46 (4.60–19.43)	<0.001		7.81 (2.87–21.24)	<0.001
≥ 135 mmol/L		1.00 (Reference)			1.00 (Reference)	
Microscopic hematuria	162			153		
Yes		10.51 (4.74–23.28)	<0.001		11.45 (3.76–34.82)	<0.001
No		1.00 (Reference)			1.00 (Reference)	
Time from bite to receiving antivenom (hours)	156			153		
≥ 2		2.45 (1.20–5.02)	0.014		7.98 (2.06–30.92)	0.003
< 2		1.00 (Reference)			1.00 (Reference)	

*Abbreviations*: *CI* confidence interval, *DIC* disseminated intravascular coagulation, *OR* odds ratio, *WBC* white blood cell

Comparison of clinical parameters, laboratory findings, management characteristics, and outcomes among 50 patients with AKI during hospitalization and 78 patients without AKI are shown in Additional file 1: Tables S10, S11, and S12. Clinical parameters, laboratory findings, and management characteristics possibly affecting renal function are shown in Table 7. Patients with the parameters of overt DIC (*p* = 0.007), presence of capillary leakage (*p* < 0.001), serum creatine kinase >500 IU/L (*p* < 0.001), serum sodium <135 mmol/L (*p* < 0.001), and who received a total dose of antivenom >160 mL (*p* = 0.016) were more likely to develop AKI during hospitalization (Table 7).

**Table 7:** Clinical parameters, laboratory findings, and management characteristics of 128 adults bitten by *Viperidae* or presenting the clinical syndrome of *Viperidae* (50 patients who developed acute kidney injury during hospitalization and 78 patients without acute kidney injury)

Characteristics	All	With acute kidney injury, *n* (%)	Without acute kidney injury, *n* (%)	*p* value
Overt DIC				
Yes	72	36 (50.0)	36 (50.0)	0.007
No	56	14 (25.0)	42 (75.0)	
Capillary leakage				
Yes	14	12 (85.7)	2 (14.3)	<0.001
No	114	38 (33.3)	76 (66.7)	
Creatine kinase (IU/L)				
> 500	40	28 (70.0)	12 (30.0)	<0.001
≤ 500	86	20 (23.3)	66 (76.7)	
Serum sodium (mmol/L)				
< 135	45	29 (64.4)	16 (35.6)	<0.001
≥ 135	83	21 (25.3)	62 (74.7)	
Total dose of antivenom (mL)				
> 160	31	19 (61.3)	12 (38.7)	0.016
≤ 160	90	31 (34.4)	59 (65.6)	

*Abbreviation*: *DIC* disseminated intravascular coagulation

These parameters were also found to be associated with AKI during hospitalization using a univariate logistic regression model (Table 8). In a multivariate logistic regression model, the following parameters were independently associated with AKI during hospitalization: presence of capillary leakage (odds ratio [OR]: 6.30; 95% CI: 1.13–35.22; *p* = 0.036), serum creatine kinase >500 IU/L (OR: 4.80, 95% CI: 1.86–12.37; *p* = 0.001), and serum sodium <135 mmol/L (OR: 4.27, 95% CI: 1.74–10.45; *p* = 0.001) (Table 8).

**Table 8:** Univariate and multivariate analysis of clinical and laboratory parameters of adults bitten by *Viperidae* or presenting the clinical syndrome of *Viperidae* for identifying acute kidney injury during hospitalization

Characteristic	Univariate analysis			Multivariate analysis		
	*n*	OR (95% CI)	*p* value	*n*	OR (95% CI)	*p* value
Overt DIC	128					
Yes		3.00 (1.40–6.42)	0.005			
No		1.00 (Reference)				
Capillary leakage	128			119		
Yes		12.00 (2.56–56.36)	0.002		6.30 (1.13–35.22)	0.036
No		1.00 (Reference)			1.00 (Reference)	
Creatine kinase (IU/L)	126			119		
> 500		7.70 (3.32–17.86)	<0.001		4.80 (1.86–12.37)	0.001
≤ 500		1.00 (Reference)			1.00 (Reference)	
Serum sodium (mmol/L)	128			119		
< 135 mmol/L		5.35 (2.44–11.74)	<0.001		4.27 (1.74–10.45)	0.001
≥ 135 mmol/L		1.00 (Reference)			1.00 (Reference)	
Total dose of antivenom (mL)	121					
> 160		3.01 (1.30–7.00)	0.010			
≤ 160		1.00 (Reference)				

*Abbreviations*: *CI* confidence interval, *DIC* disseminated intravascular coagulation, *OR* odds ratio

### Evaluation of electrolyte abnormalities among patients with normal renal function at presentation

In our study, electrolyte abnormalities, particularly hyponatremia (serum sodium <135 mmol/L) and hypokalemia (serum potassium <3.5 mmol/L), were common renal manifestations among the 258 patients with snake envenomation. In addition, hyponatremia was found to be an independent factor associated with AKI. Thus, evaluation of renal tubular function was performed among patients with snakebites from *Viperidae* who had normal renal function. At presentation, there were 128 patients who had been bitten by *Viperidae* or demonstrated the clinical syndrome of *Viperidae* envenoming with normal renal function. The subsequent electrolyte abnormalities observed in these patients included hyponatremia (45/128 patients, 35.2%), hypocalcemia (33/126 patients, 26.2%), hypokalemia (32/128 patients, 25.0%), hyperphosphatemia (21/126 patients, 16.7%), hypophosphatemia (12/126 patients, 9.5%), metabolic acidosis (7/128 patients, 5.5%), hypernatremia (4/128 patients, 3.1%), hyperkalemia (4/128 patients, 3.1%), and hypercalcemia (2/126 patients, 1.6%).

**Fig. 3:**
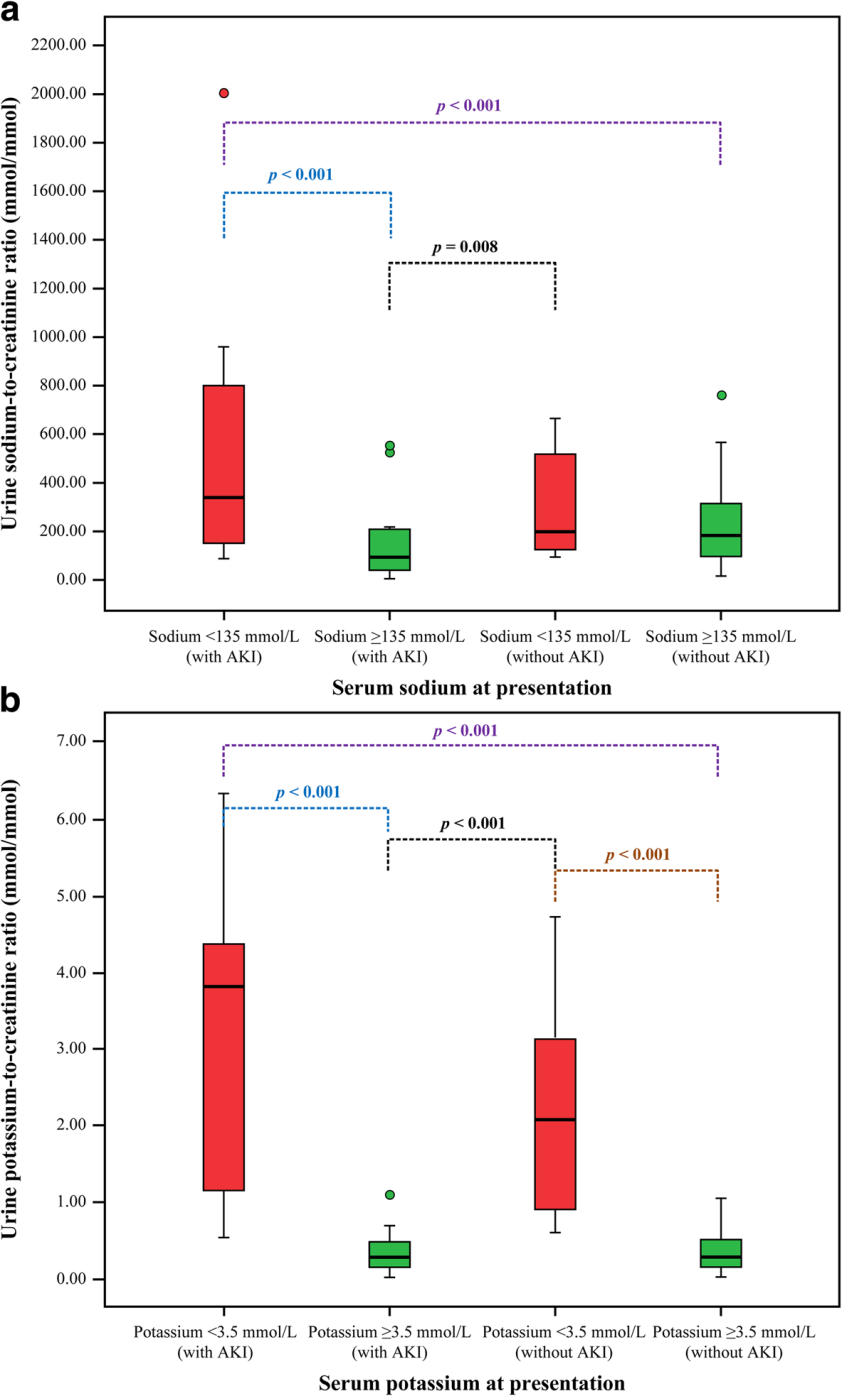
Urine electrolyte-to-creatinine ratios and serum electrolytes among 128 patients with normal kidney function at presentation, stratified by the development of acute kidney injury during hospitalization. **a** Urine sodium-to-creatinine ratio (mmol/mmol) among patients with serum sodium <135 mmol/L and those with serum sodium ≥135 mmol/L. **b** Urine potassium-to-creatinine ratio (mmol/mmol) among patients with serum potassium <3.5 mmol/L and those with serum potassium ≥3.5 mmol/L. Data are presented as *box* and *whisker plots* with median (*horizontal line*), interquartile range (*box*), and maximum value within 1.5 of interquartile range (*whiskers*)

**Fig. 4:**
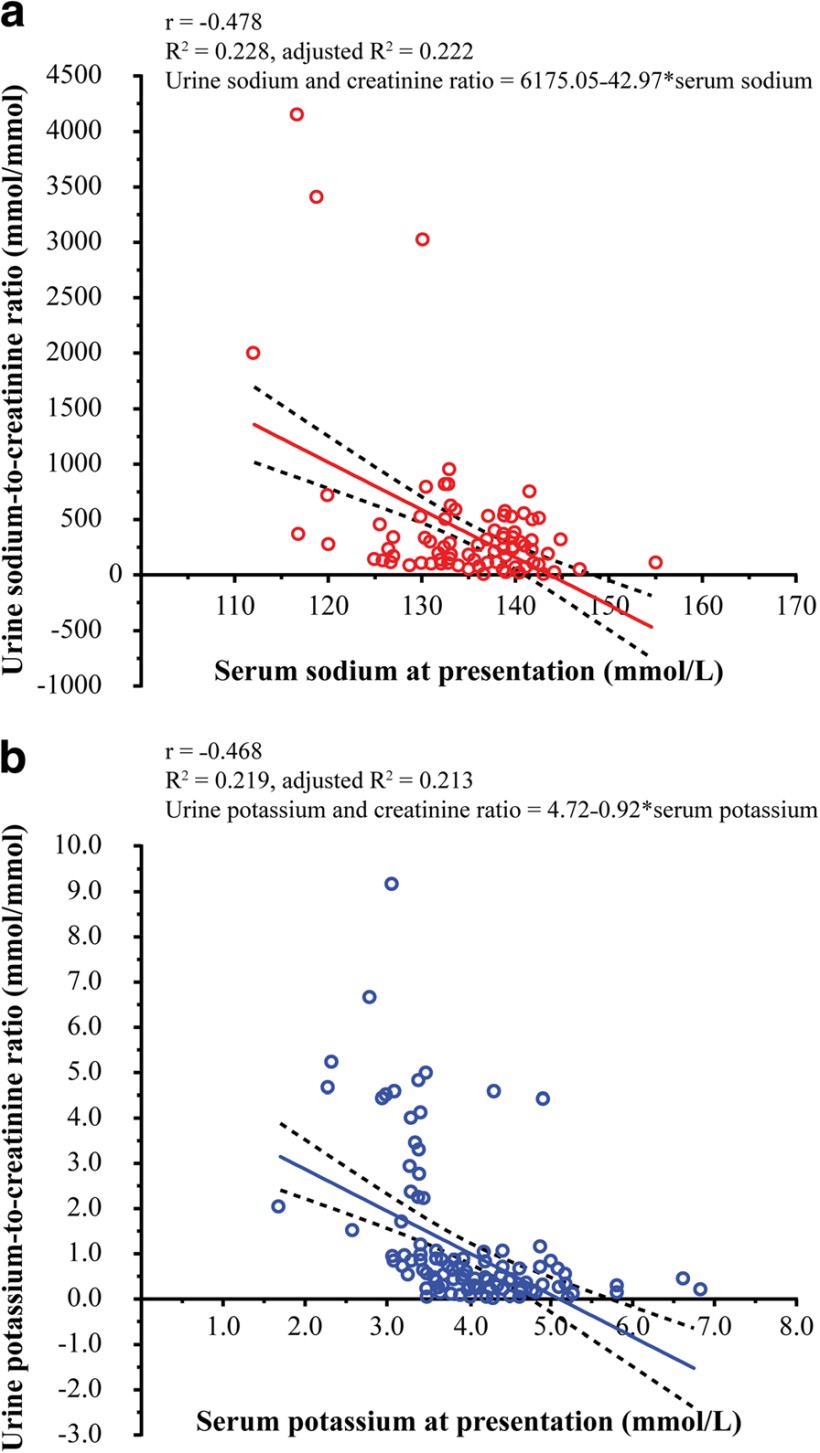
Linear regression analysis of urine electrolyte-to-creatinine ratios and levels of serum electrolytes. **a** Urine sodium-to-creatinine ratio (mmol/mmol) *plotted* against serum sodium (mmol/L) levels among 128 patients with normal renal function. **b** Urine potassium-to-creatinine ratio (mmol/mmol) *plotted* against serum potassium (mmol/L) levels among 128 patients with normal renal function

Patients with hyponatremia had significantly higher urine sodium-to-creatinine ratio than those with serum sodium ≥135 mmol/L [median (IQR) 291.5 (139.7–610.6) mmol/mmol vs. 173.2 (72.3–314.9) mmol/mmol, *p* < 0.001]. When these patients were subdivided into those who developed AKI during hospitalization and those who did not, 29 patients (22.7%) with hyponatremia developed AKI, 21 (16.4%) with serum sodium ≥135 mmol/L developed AKI, 16 (12.5%) with hyponatremia did not develop AKI, and 62 (48.4%) with serum sodium ≥135 mmol/L did not develop AKI. A significant difference in median urine sodium-to-creatinine ratio was observed across the groups (Kruskal-Wallis test, chi-squared = 21.285, degrees of freedom = 3, *p* < 0.001). Post-hoc analysis using the Mann-Whitney *U* test with Bonferroni correction indicated that the median [IQR] urine sodium-to-creatinine ratio of patients with hyponatremia who developed AKI during hospitalization (343.8 [154.0–817.3]) was significantly higher than that of patients with serum sodium ≥135 mmol/L who developed AKI during hospitalization (96.0 [35.7–219.7], *p* < 0.001) as well as that of patients with serum sodium ≥135 mmol/L who did not develop AKI (183.8 [101.3–319.5], *p* < 0.001). However, urine sodium-to-creatinine ratio did not significantly differ between hyponatremic patients who developed AKI during hospitalization (343.8 [154.0–817.3]) and those who did not (198.9 [124.9 − 529.3], *p* = 0.083). The median [IQR] urine sodium-to-creatinine ratio of patients with hyponatremia who did not develop AKI (198.9 [124.9–529.3]) was significantly higher than that of patients with serum sodium ≥135 mmol/L who developed AKI during hospitalization (96.0 [35.7–219.7], *p* = 0.008), but not significantly different from that of patients with serum sodium ≥135 mmol/L who did not develop AKI (183.8 [101.3–319.5], *p* = 0.189) (Fig. 3a). There was a significant regression of urine sodium-to-creatinine ratio to serum sodium (urine sodium-to-creatinine ratio = 6175.05–42.97*serum sodium, F_(1127)_ = 36.075, *p* < 0.001), whereby 22.2% of variation could be explained by the regression line (*R* ^2^ = 0.228, adjusted *R* ^2^ = 0.222). Serum sodium and urine sodium-to-creatinine ratio were also correlated (*r* = –0.478, *p* < 0.001) (Fig. 4a).

Patients with hypokalemia had significantly higher urine potassium-to-creatinine ratio than those with serum potassium ≥3.5 mmol/L [median (IQR) 2.2 (0.9–4.3) mmol/mmol vs. 0.3 (0.2–0.5) mmol/mmol, *p* < 0.001). When these patients were subdivided into those who developed AKI during hospitalization and those who did not, 13 patients (10.1%) with hypokalemia had AKI, 37 (28.9%) with serum potassium ≥3.5 mmol/L had AKI, 18 (14.1%) with hypokalemia did not have AKI, and 60 (46.9%) with serum potassium ≥3.5 mmol/L did not have AKI. A significant difference in median urine potassium-to-creatinine ratio was observed across the groups (Kruskal-Wallis test, chi-squared = 61.615, degrees of freedom = 3, *p* < 0.001). Post-hoc analysis using the Mann-Whitney *U* test with Bonferroni correction indicated that the median [IQR] urine potassium-to-creatinine ratio of patients with hypokalemia who developed AKI during hospitalization (3.8 [1.0–4.5]) was significantly higher than that of patients with serum potassium ≥3.5 mmol/L who developed AKI during hospitalization (0.3 [0.2–0.5], *p* < 0.001) and that of patients with serum potassium ≥3.5 mmol/L who did not develop AKI (0.3 [0.2–0.5], *p* < 0.001). However, urine potassium-to-creatinine ratio did not significantly differ between hypokalemic patients who developed AKI during hospitalization (3.8 [1.0–4.5]) and those who did not (2.1 [0.9–3.2], *p* = 0.242). The median [IQR] urine potassium-to-creatinine ratio of patients with hypokalemia who did not develop AKI (2.1 [0.9–3.2]) was significantly higher than that of patients with serum potassium ≥3.5 mmol/L who developed AKI during hospitalization (0.3 [0.2–0.5], *p* < 0.001) and that of patients with serum potassium ≥3.5 mmol/L who did not develop AKI (0.3 [0.2–0.5], *p* < 0.001) (Fig. 3b). There was a significant regression of urine potassium-to-creatinine ratio to serum potassium (urine potassium-to-creatinine ratio = 4.72–0.92*serum potassium, F_(1127)_ = 35.056, *p* < 0.001), whereby 21.3% of variation could be explained by the regression line (*R* ^2^ = 0.219, adjusted *R* ^2^ = 0.213). Serum potassium and urine potassium-to-creatinine ratio were also correlated (*r* = –0.468, *p* < 0.001) (Fig. 4b).

Serum magnesium and fractional excretion of magnesium were measured in 28 patients with hypokalemia. The median (IQR) of serum magnesium was 2.0 (1.8–2.2) mg/dL, with fractional magnesium excretion of 2.4 (0.7–4.9)%. Of 28 patients with hypokalemia, hypomagnesemia was observed in 6 patients (21.4%) and serum magnesium ≥1.7 mg/dL in 22 patients (78.6%). The median (IQR) fractional excretion of magnesium was 6.4 (2.9–8.6)% among patients with hypomagnesemia and 2.0 (0.6–3.7)% among those with serum magnesium ≥1.7 mg/dL (*p* = 0.017). All patients with hypomagnesemia had fractional excretion of magnesium >1.0%. Of 22 patients with serum magnesium ≥1.7 mg/dL, 8 (36.4%) had fractional excretion of magnesium <1%, and 14 (63.6%) had fractional excretion of magnesium >1.0%.

The median (IQR) fractional excretion of calcium was 0.8 (0.2–4.0)% among patients with hypocalcemia, and fractional excretion of phosphate was 5.4 (2.3–6.7)% among patients with hypophosphatemia. No patients with hypophosphatemia had fractional excretion of phosphate >15.0%, and no patients with hypocalcemia had fractional excretion of calcium >20.0%.

## Discussion

Snakebite envenomation is one of the common causes of cAKI in tropical countries, particularly in Southeast Asia [1, 2, 4]. The reported incidence of renal involvement with snakebite envenomation ranges from 1.4–28.0% [25, 26]. Renal involvement including proteinuria, hematuria, pigmenturia, and AKI is commonly observed among patients with snakebites from snakes of the family *Viperidae* [5–8]. Hemotoxic and myotoxic venom, particularly from Russell’s viper and sea snake, are common causes of renal involvement [7, 8]. Similarly, in our study, the majority of patients were bitten by Russell’s viper (84.5%) and clinical renal manifestations were frequent among patients with snakebite envenomation, particularly reduced urine volume (49.6%), renal tenderness (44.2%), and urine protein-to-creatinine ratio ≥1 (60.2%). In Myanmar, proteinuria >1 g/24 h was previously reported in 50.0% of patients with Russell’s viper bites [27] and hematuria in 35.0% of patents with hemotoxic snakebites or the occurrence of glomerulonephritis after snakebite envenomation [7, 8]. Previous reports showed that Russell’s viper can cause both intravascular hemolysis and rhabdomyolysis, which is induced by phospholipase A_2_ in snake venom [7, 8].

In our cohort, 140 patients (54.3%) had sAKI according to the criteria of the KDIGO clinical practice guidelines [22]. In previous reports, sAKI was observed in 8.0–43.0% of patients with snakebite envenomation. The higher observed rate in the present study might be due to differences in criteria for AKI diagnosis, snake species, snake venom potency, and genetic variation of the victims [9–15, 19]. In our study, 62.9% of AKI patients had sAKI at presentation, and the rest (37.1%) developed sAKI during hospitalization. Multivariate logistic regression analysis revealed the following parameters to be independently associated with sAKI: bites from snakes of the *Viperidae* family or presenting the clinical syndrome of *Viperidae* envenoming, leukocytosis, overt DIC, rhabdomyolysis, hyponatremia, glomerulonephritis, and duration from snakebite to receipt of antivenom ≥2 h. Among patients bitten by *Viperidae*, the presence of hypotension, rhabdomyolysis, hyponatremia, glomerulonephritis, and duration from snakebite to receipt of antivenom ≥2 h were independently associated with sAKI at presentation. In addition, patients bitten by *Viperidae* who presented with capillary leakage, rhabdomyolysis, and hyponatremia were at increased risk for the development of sAKI during hospitalization. Previous studies reported similar factors associated with the sAKI, including receipt of antivenom >2 h after snakebite, cellulitis, hypotension, and rhabdomyolysis [9, 10, 13]. A previous case series from Taiwan showed that early specific antivenom treatment within 3 to 6 h after snake envenoming could restore coagulation abnormalities in 1 to 2 days and was effective in reducing the severity of renal damage [28].

Our finding that leukocytosis was associated with sAKI is consistent with a previous experimental study in which leukocytosis was observed after injection of phospholipase A_2_ and metalloprotease from Russell’s viper venom, which resulted in the elevation of interleukin-6, tumor necrosis factor-α, and prostaglandin E_2_ leading to increased renal vascular resistance and decreased blood pressure [29]. The observation of glomerular proteinuria and microscopic hematuria in our study suggested the occurrence of glomerulonephritis among patients with sAKI. A previous study from Myanmar also showed that 50% of patients with Russell’s viper bites had proteinuria >1 g/24 h [27].

We found that a substantial number of patients with sAKI had hyponatremia, and patients with sAKI had significantly higher fractional excretion of sodium than those without sAKI, indicating the occurrence of renal tubular damage among patients with sAKI, which is consistent with a previous study from Myanmar reporting increased levels of beta-2 microglobulin and N-acetyl glucosaminidase in urine among patients with sAKI from Russell’s viper bites, also indicating renal tubular damage [27]. A previous report from India also showed that the renal pathology of AKI patients with *Viperidae* bites involved acute tubular necrosis, which might be due to the direct toxicity of the snake venom [30]. However, our patients with sAKI had significantly lower fractional excretion of urea (28.5%) than those without sAKI (42.3%), as well as serum urea-to-creatinine ratio >20 and urine specific gravity of 1.020, indicating renal ischemia that might contribute to the development of sAKI. A previous study from Myanmar also showed that plasma renin concentrations were highly elevated among patients with sAKI from Russell’s viper bites, suggesting the occurrence of renal ischemia in these patients [27]. A previous experimental study revealed massive fibrin deposition in glomerular capillaries, proximal and distal tubular necrosis, and hemolyzed red blood cell casts in the renal tubules of rats subjected to intravascular injection of snake venom, indicating that DIC contributed to renal ischemia in sAKI [31].

Furthermore, snake venom, particularly *Viperidae* snake venom containing proteolytic enzymes, can destruct the endothelium and basal membrane of capillaries. Thus, capillary permeability increases, albumins escape to the perivascular space, tissue oncotic pressure increases, and plasma oncotic pressure decreases, resulting in a shift of fluid balance from the intravascular to interstitial space, or capillary leakage. The decrease in intravascular volume may be sufficiently severe to compromise circulation, resulting in shock [32]. In cases of severe envenomation, snake venom can induce the release of autopharmacological vasoactive substances, particularly bradykinin, resulting in vasodilatation and myocardial depression, and consequently reduce myocardial contractility [32]. Therefore, our findings support the pathophysiology of sAKI as multifactorial, including hemodynamic changes, direct toxicity of snake venom, immunologic reaction and pigmenturia [33]. This pathophysiology would explain pathological observation of tubular necrosis, mesangiolysis, cortical necrosis, vasculitis, glomerulonephritis, interstitial nephritis, and renal infarction after snakebite envenomation [33].

In our study, 49.3% of patients with sAKI required RRT, which is within the range of previous reports (15.0–55.0%) [9–11, 13]. The fatality rate was 19.3%, which is also in the reported range (8.0–39.0%) among patients with snakebite envenomation [9–11, 13, 14]. When clinical parameters and management of patients with sAKI were compared between patients who died and those who survived, patients who died were more likely to develop shock, have pulmonary complications, and receive management in the intensive care unit. However, the proportions of patients who received RRT and peritoneal dialysis as a dialysis method were similar between those who died and survived. The adequacy of dialysis determined by weekly Kt/V was less than 2.1 among AKI patients who died (median: 1.9, IQR: 1.1–3.0), but not among those who survived (median: 2.7, IQR: 2.2–3.9). However, there was no statistically significant difference between the groups, and it is probable that a small number of patients who died (*n* = 8) were evaluated for weekly Kt/V. According to the ISPD guideline for peritoneal dialysis in AKI, the target weekly Kt/V of 3.5 provides outcomes comparable to that of daily hemodialysis. However, a target weekly Kt/V of 2.1 may be acceptable [23].

In our study, electrolyte abnormalities were common among patients with snakebite envenomation. Patients with snakebite envenomation by a member of the *Viperidae* family who had hyponatremia at presentation and developed AKI during hospitalization had a significantly higher urine sodium-to-creatinine ratio. Our findings also indicated a significant negative correlation between urine sodium-to-creatinine ratio and serum sodium (*r* = –0.478), suggesting that hyponatremia among patients with *Viperidae* snakebites resulted from the renal tubular loss of sodium. Similarly, patients with hypokalemia who developed AKI during hospitalization had a significantly higher urine potassium-to-creatinine ratio. The significant negative correlation of urine potassium-to-creatinine ratio and serum potassium (*r* = –0.468) suggests that hypokalemia among patients with *Viperidae* bites resulted from the renal tubular loss of potassium. Moreover, hypomagnesemia was observed in 21.4% of patients with hypokalemia, all of whom had urinary loss of magnesium. Our findings suggested that the renal tubular loss of electrolytes including sodium, potassium and magnesium might be caused by the direct toxicity of snake venom from the *Viperidae* family. A previous experimental study of Russell’s viper venom demonstrated decreased renal tubular absorption of sodium in proximal and distal renal tubules resulting in increased fractional excretion of sodium due to Na + −K + −ATPase activity inhibition of renal tubules in both the renal cortex and medulla [34].

None of the patients with hypocalcemia had fractional excretion of calcium >20.0% and none of the patients with hypophosphatemia had fractional excretion of phosphate >15.0%, indicating extrarenal causes of hypocalcemia and hypophosphatemia. By contrast, hyperphosphatemia was more commonly observed compared to hypophosphatemia among patients with snakebite envenomation in our study. Thus, hypocalcemia might be caused by the process of intravascular thrombosis following the occurrence of DIC and rhabdomyolysis, and the occurrence of rhabdomyolysis would result in hyperphosphatemia among patients with snakebite envenomation [35, 36]. However, the possibility that hyperphosphatemia might occur secondary to AKI snake envenoming cannot be excluded [37].

### Strengths and limitations

Our study has some limitations. First, all the data came from three academic tertiary care hospitals, which limits the generalizability of the study findings. Second, the snake could be identified in only 67.4% of cases. In case of unidentified snake bites, patient management relied on the clinical syndromes of snakebites defined in the WHO 2010 guidelines. However, our study was conducted as a prospective observational study in order to reduce bias, and the number of study patients achieved the required sample size for adequate statistical power. In addition, this study was the first to demonstrate the occurrence of impaired renal tubular function among patients with normal renal function who received bites from snake of the family *Viperidae* or having clinical syndrome of *Viperidae* envenoming. 

## Conclusion

Renal manifestations among patients with snake envenomation included reduce urine volume, renal tenderness, proteinuria, hematuria, electrolytes abnormalities, and AKI, and sAKI was a common and significant complication. The factors associated with sAKI included bites from snakes of the family *Viperidae* or presenting the clinical syndrome of *Viperidae* envenoming, duration from bite to receipt of antivenom ≥2 h, leukocytosis, overt DIC, rhabdomyolysis, hyponatremia and presence of microscopic hematuria. Our findings support the hypothesis of multifactorial involvement in the pathogenesis of sAKI. After sustaining *Viperidae* bites, a significant number of patients with normal renal function developed proximal and distal renal tubular dysfunction. These findings might help clinicians to provide optimal management of patients who are at risk for the development of sAKI in order to reduce the incidence of cAKI in tropical countries in the future.

## Additional file

Additional file 1: Table S1. Definitions of abnormal urine findings and electrolyte abnormalities. **Table S2.** Definitions of clinical parameters of snake envenoming according to the WHO 2010 guidelines. **Table S3.** Baseline characteristics and pre-hospital management among 258 adults with snakebite envenomation, Yangon, Myanmar, 2015–2016. **Table S4.** Clinical presentation among 258 adults with snakebite envenomation, Yangon, Myanmar, 2015–2016. **Table S5.** Laboratory parameters at presentation among 258 adults with snakebite envenomation, Yangon, Myanmar, 2015–2016. **Table S6.** Management and outcomes among 258 adults with snakebite envenomation, Yangon, Myanmar, 2015–2016. **Table S7.** Clinical parameters among 164 adults bitten by *Viperidae* or presenting the clinical syndrome of *Viperidae* (86 patients with acute kidney injury at presentation and 78 patients without acute kidney injury). **Table S8.** Laboratory parameters among 164 adults bitten by *Viperidae* or presenting the clinical syndrome of *Viperidae* (86 patients with acute kidney injury at presentation and 78 patients without acute kidney injury). **Table S9.** Management and outcomes among 164 adults bitten by *Viperidae* or presenting the clinical syndrome of *Viperidae* (86 patients with acute kidney injury at presentation and 78 patients without acute kidney injury). **Table S10.** Clinical parameters among 128 adults bitten by *Viperidae* or presenting the clinical syndrome of *Viperidae* (50 patients who developed acute kidney injury during hospitalization and 78 patients without acute kidney injury). **Table S11.** Laboratory parameters among 128 adults bitten by *Viperidae* or presenting the clinical syndrome of *Viperidae* (50 patients who developed acute kidney injury during hospitalization and 78 patients without acute kidney injury). **Table S12.** Management and outcomes among 128 adults bitten by *Viperidae* or presenting the clinical syndrome of *Viperidae* (50 patients who developed acute kidney injury during hospitalization and 78 patients without acute kidney injury). The supplementary tables provide additional data to help interpret the results of the study. (DOCX 92 kb)

## Abbreviations

20WBCT: 20-min whole blood clotting test
AKI: Acute kidney injury
ALT: Alanine aminotransferase
aPTT: Activated partial thromboplastin time
ARDS: Acute respiratory distress syndrome
AST: Aspartate aminotransferase
cAKI: Community-acquired acute kidney injury
CI: Confidence interval
DIC: Disseminated intravascular coagulation
ECG: Electrocardiography
Fe: Fractional excretion
HPF: high power field
ICU: Intensive care unit
IGH: Insein General Hospital
INR: International normalized ratio
IQR: Interquartile range
ISTH: International Society for Thrombosis and Haemostasis
NOGH: North Okkalapa General Hospital
OR: Odds ratio
PLT: Platelet
PT: Prothrombin time
RRT: Renal replacement therapy
sAKI: Snakebite related acute kidney injury
SBP: Systolic blood pressure
STROBE: Standards for the Reporting of Observation Studies in Epidemiology
TGH: Thingangyun Sanpya General Hospital
WBC: White blood cell
WHO: World Health Organization
