# Supplementary material for: Clinical and laboratory parameters associated with acute kidney injury in patients with snakebite envenomation: a prospective observational study from Myanmar
Source: BMC Nephrol. 2017 Mar 16;18:92. doi: 10.1186/s12882-017-0510-0 (PMC5353953; doi:10.1186/s12882-017-0510-0)
Supplement: Additional file 1: Table S1. — Definitions of abnormal urine findings and electrolyte abnormalities. Table S2. Definitions of clinical parameters of snake envenoming according to the WHO 2010 guidelines. Table S3. Baseline characteristics and pre-hospital management among 258 adults with snakebite envenomation, Yangon, Myanmar, 2015–2016. Table S4. Clinical presentation among 258 adults with snakebite envenomation, Yangon, Myanmar, 2015–2016. Table S5. Laboratory parameters at presentation among 258 adults with snakebite envenomation, Yangon, Myanmar, 2015–2016. Table S6. Management and outcomes among 258 adults with snakebite envenomation, Yangon, Myanmar, 2015–2016. Table S7. Clinical parameters among 164 adults bitten by Viperidae or presenting the clinical syndrome of Viperidae (86 patients with acute kidney injury at presentation and 78 patients without acute kidney injury). Table S8. Laboratory parameters among 164 adults bitten by Viperidae or presenting the clinical syndrome of Viperidae (86 patients with acute kidney injury at presentation and 78 patients without acute kidney injury). Table S9. Management and outcomes among 164 adults bitten by Viperidae or presenting the clinical syndrome of Viperidae (86 patients with acute kidney injury at presentation and 78 patients without acute kidney injury). Table S10. Clinical parameters among 128 adults bitten by Viperidae or presenting the clinical syndrome of Viperidae (50 patients who developed acute kidney injury during hospitalization and 78 patients without acute kidney injury). Table S11. Laboratory parameters among 128 adults bitten by Viperidae or presenting the clinical syndrome of Viperidae (50 patients who developed acute kidney injury during hospitalization and 78 patients without acute kidney injury). Table S12. Management and outcomes among 128 adults bitten by Viperidae or presenting the clinical syndrome of Viperidae (50 patients who developed acute kidney injury during hospitalization and 78 patients without acute kidney inju [file 12882_2017_510_MOESM1_ESM.docx]

**Additional File 1**

**Supplementary Tables**

**Clinical and laboratory parameters associated with acute kidney injury in patients with snakebite envenomation: A prospective observational study from Myanmar**

Kyi-Phyu-Aye, Vipa Thanachartwet, Chit-Soe, Varunee Desakorn, Khin-Thida-Thwin, Supat Chamnanchanunt, Duangjai Sahassananda, Thanom Supaporn, Visith Sitprija

**Table S1.** Definitions of abnormal urine findings and electrolyte abnormalities

| Parameters | Definitions |
| --- | --- |
| Abnormal urine findings |  |
| Microscopic hematuria | RBC in a spun urine ≥3 cells/HPF |
| Leukocyturia | WBC in a spun urine ≥5 cells/HPF |
| Pigmenturia | Positive heme from urine dipstick with RBC in a spun urine <3 cells/HPF |
| Electrolyte abnormalities |  |
| Hyponatremia | Serum sodium <135 mmol/L |
| Hypernatremia | Serum sodium >145 mmol/L |
| Hypokalemia | Serum potassium <3.5 mmol/L |
| Hyperkalemia | Serum potassium >5.5 mmol/L |
| Hypomagnesemia | Serum magnesium <1.7 mg/dL |
| Hypocalcemia | Serum calcium <8.0 mg/dL |
| Hypercalcemia | Serum calcium ≥10.5 mg/dL |
| Hypophosphatemia | Serum phosphate ≤2.5 mg/dL |
| Hyperphosphatemia | Serum phosphate ≥5.5 mg/dL |
| Metabolic acidosis | Serum bicarbonate <15 mmol/L |
| Metabolic alkalosis | Serum bicarbonate ≥40 mmol/L |

HPF, high-power field; RBC, red blood cell; WBC, white blood cell.

**Table S2.** Definitions of clinical parameters of snake envenoming according to the WHO 2010 guidelines

| Clinical parameters | Definitions |
| --- | --- |
| Non-specific symptoms and signs | nausea, vomiting, malaise, weakness, abdominal pain, drowsiness and prostration |
| Local symptoms and signs | fang marks, local pain, bleed per fang mark, bruising, lymphangitis, tender lymphadenitis, local swelling, blistering, local infection including cellulitis and abscess, skin necrosis and gangrene |
| Degree of local swelling | swelling confined to the joint adjacent to the wound (Grade I)  swelling extended beyond the first adjacent joint, but not beyond the second joint (Grade II)  swelling extended the second joint, but not beyond the third joint (Grade III)  swelling beyond the third joint (Grade IV) |
| Systemic symptoms and signs |  |
| Cardiovascular manifestations | visual disturbances, dizziness, collapse, conjunctival edema, hypotension and abnormal ECG |
| Hemostatic manifestations | local bleeding, spontaneous systemic bleeding, incoagulable blood by 20WBCT and DIC |
| Neurological manifestations | muscle weakness, bulbar palsy, ptosis, external opthalmoplegia and paresthesia |
| Renal manifestations | dark color urine, renal tenderness, reduce urine volume and acute kidney injury |

ECG, electrocardiography; DIC, disseminated intravascular coagulation; 20WBCT, 20-minute whole blood clotting test; WHO, World Health Organization.

**Table S3.** Baseline characteristics and pre-hospital management among 258 adults with snakebite envenomation, Yangon, Myanmar, 2015−2016

| Characteristics | All | With acute kidney injury, n (%) | Without acute kidney injury, n (%) | *p* value |
| --- | --- | --- | --- | --- |
| *Baseline characteristics* |  |  |  |  |
| Study site |  |  |  |  |
| TGH | 229 | 122 (53.3) | 107 (46.7) | 0.485 |
| IGH and NOGH* | 29 | 18 (62.1) | 11 (37.9) |  |
| Age (years), median (IQR) | 258 | 30.0 (23.0˗41.0) | 34.5 (25.0˗44.0) | 0.119 |
| Sex |  |  |  |  |
| Male | 203 | 114 (56.2) | 89 (43.8) | 0.307 |
| Female | 55 | 26 (47.3) | 29 (52.7) |  |
| Race |  |  |  |  |
| Myanmar | 222 | 116 (52.3) | 106 (47.7) | 0.153 |
| Non Myanmar | 36 | 24 (66.7) | 12 (33.3) |  |
| Residence |  |  |  |  |
| Yangon city | 188 | 121 (64.4) | 67 (35.6) | <0.001 |
| Outside Yangon city | 70 | 19 (27.1) | 51 (72.9) |  |
| Place of bite |  |  |  |  |
| Field | 187 | 116 (62.0) | 71 (38.0) | <0.001 |
| House or road | 71 | 24 (33.8) | 47 (66.2) |  |
| Anatomical site of bite |  |  |  |  |
| Lower extremities | 205 | 129 (62.9) | 76 (37.1) | <0.001 |
| Upper extremities | 53 | 11 (20.8) | 42 (79.2) |  |
| Snake identification |  |  |  |  |
| Yes | 174 | 89 (51.1) | 85 (48.9) | 0.190 |
| No | 84 | 51 (60.7) | 33 (39.3) |  |
| *Bites from Viperidae* or presenting clinical syndrome of *Viperidae* |  |  |  |  |
| Yes | 214 | 136 (63.6) | 78 (36.4) | <0.001 |
| No | 44 | 4 (9.1) | 40 (90.9) |  |
| *Pre-hospital management* |  |  |  |  |
| Applied tourniquet |  |  |  |  |
| Yes | 210 | 122 (58.1) | 88 (41.9) | 0.015 |
| No | 48 | 18 (37.5) | 30 (62.5) |  |
| Mobilization |  |  |  |  |
| Yes | 185 | 101 (54.6) | 84 (45.4) | 0.975 |
| No | 73 | 39 (53.4) | 34 (46.6) |  |
| Cleaning |  |  |  |  |
| Yes | 38 | 18 (47.4) | 20 (52.6) | 0.455 |
| No | 220 | 122 (55.5) | 98 (44.5) |  |
| Wound incision or suction |  |  |  |  |
| Yes | 32 | 23 (71.9) | 9 (28.1) | 0.052 |
| No | 226 | 117 (51.8) | 109 (48.2) |  |
| Traditional healer |  |  |  |  |
| Yes | 27 | 20 (74.1) | 7 (25.9) | 0.048 |
| No | 231 | 120 (51.9) | 111 (48.1) |  |

IGH, Insein General Hospital; IQR, interquartile range; NOGH, North Okkalapa General Hospital; TGH, Thingangyun Sanpya General Hospital.

*The number of patients treated at IGH and NOGH was too low for individual comparison; therefore, these patients were merged into one group.

**Table S4.** Clinical presentation among 258 adults with snakebite envenomation, Yangon, Myanmar, 2015−2016

| Characteristics | All | With acute kidney injury, n (%) | Without acute kidney injury, n (%) | *p* value |
| --- | --- | --- | --- | --- |
| *Local symptoms and signs* |  |  |  |  |
| Fang marks |  |  |  |  |
| Yes | 249 | 134 (53.8) | 115 (46.2) | 0.515 |
| No | 9 | 6 (66.7) | 3 (33.3) |  |
| Pain |  |  |  |  |
| Yes | 242 | 134 (55.4) | 108 (44.6) | 0.258 |
| No | 16 | 6 (37.5) | 10 (62.5) |  |
| Tender lymphadenitis |  |  |  |  |
| Yes | 175 | 120 (68.6) | 55 (31.4) | <0.001 |
| No | 83 | 20 (24.1) | 63 (75.9) |  |
| Local swelling |  |  |  |  |
| Grades II-IV | 167 | 111 (66.5) | 56 (33.5) | <0.001 |
| Grades 0-I | 91 | 29 (31.9) | 62 (68.1) |  |
| Skin infection |  |  |  |  |
| Yes | 75 | 47 (62.7) | 28 (37.3) | 0.110 |
| No | 183 | 93 (50.8) | 90 (49.2) |  |
| Skin necrosis or gangrene |  |  |  |  |
| Yes | 48 | 30 (62.5) | 18 (37.5) | 0.267 |
| No | 210 | 110 (52.4) | 100 (47.6) |  |
| Bruising |  |  |  |  |
| Yes | 35 | 31 (88.6) | 4 (11.4) | <0.001 |
| No | 223 | 109 (48.9) | 114 (51.1) |  |
| Blistering |  |  |  |  |
| Yes | 24 | 16 (66.7) | 8 (33.3) | 0.287 |
| No | 234 | 124 (53.0) | 110 (47.0) |  |
| *Non-specific symptoms and signs* |  |  |  |  |
| Vomiting |  |  |  |  |
| Yes | 145 | 100 (69.0) | 45 (31.0) | <0.001 |
| No | 113 | 40 (35.4) | 73 (64.6) |  |
| Muscle pain |  |  |  |  |
| Yes | 68 | 50 (73.5) | 18 (26.6) | <0.001 |
| No | 190 | 90 (47.4) | 100 (52.6) |  |
| Abdominal pain |  |  |  |  |
| Yes | 62 | 49 (79.0) | 13 (21.0) | <0.001 |
| No | 196 | 91 (46.4) | 105 (53.6) |  |
| Prostration |  |  |  |  |
| Yes | 23 | 14 (60.9) | 9 (39.1) | 0.655 |
| No | 235 | 126 (53.6) | 109 (46.4) |  |
| *Cardiovascular presentations* |  |  |  |  |
| Visual disturbance |  |  |  |  |
| Yes | 97 | 53 (54.6) | 44 (45.4) | 1.000 |
| No | 161 | 87 (54.0) | 74 (46.0) |  |
| Abnormal ECG |  |  |  |  |
| Yes | 85 | 65 (76.5) | 20 (23.5) | <0.001 |
| No | 173 | 75 (43.4) | 98 (56.6) |  |
| Hypotension |  |  |  |  |
| Yes | 69 | 60 (87.0) | 9 (13.0) | <0.001 |
| No | 189 | 80 (42.3) | 109 (57.7) |  |
| Shock |  |  |  |  |
| Yes | 58 | 54 (93.1) | 4 (6.9) | <0.001 |
| No | 200 | 86 (43.0) | 114 (57.0) |  |
| Conjunctival edema |  |  |  |  |
| Yes | 38 | 36 (94.7) | 2 (5.3) | <0.001 |
| No | 220 | 104 (47.3) | 116 (52.7) |  |
| Pulmonary edema |  |  |  |  |
| Yes | 17 | 17 (100.0) | 0 (0) | <0.001 |
| No | 241 | 123 (51.0) | 118 (49.0) |  |
| *Hemostatic presentations* |  |  |  |  |
| Systemic bleeding |  |  |  |  |
| Yes | 92 | 84 (91.3) | 8 (8.7) | <0.001 |
| No | 166 | 56 (33.7) | 110 (66.3) |  |
| Local bleeding |  |  |  |  |
| Yes | 20 | 15 (75.0) | 5 (25.0) | 0.088 |
| No | 238 | 125 (52.5) | 113 (47.5) |  |
| *Neurological presentations* |  |  |  |  |
| Ptosis |  |  |  |  |
| Yes | 43 | 7 (16.3) | 36 (83.7) | <0.001 |
| No | 215 | 133 (61.9) | 82 (38.1) |  |
| Bulbar palsy |  |  |  |  |
| Yes | 23 | 4 (17.4) | 19 (82.6) | <0.001 |
| No | 235 | 136 (57.9) | 99 (42.1) |  |
| Ophthalmoplegia |  |  |  |  |
| Yes | 22 | 2 (9.1) | 20 (90.9) | <0.001 |
| No | 236 | 138 (58.5) | 98 (41.5) |  |
| Muscular weakness |  |  |  |  |
| Yes | 16 | 2 (12.5) | 14 (87.5) | 0.001 |
| No | 242 | 138 (57.0) | 104 (43.0) |  |
| *Renal presentations* |  |  |  |  |
| Reduce urine volume |  |  |  |  |
| Yes | 128 | 121 (94.5) | 7 (5.5) | <0.001 |
| No | 130 | 19 (14.6) | 111 (85.4) |  |
| Renal tenderness |  |  |  |  |
| Yes | 114 | 97 (85.1) | 17 (14.9) | <0.001 |
| No | 144 | 43 (29.9) | 101 (70.1) |  |
| Puffy eyelids |  |  |  |  |
| Yes | 53 | 52 (98.1) | 1 (1.9) | <0.001 |
| No | 205 | 88 (42.9) | 117 (57.1) |  |
| Dark- colored urine |  |  |  |  |
| Yes | 25 | 21 (84.0) | 4 (16.0) | 0.003 |
| No | 233 | 119 (51.1) | 114 (48.9) |  |

ECG, electrocardiography.

**Table S5.** Laboratory parameters at presentation among 258 adults with snakebite envenomation, Yangon, Myanmar, 2015−2016

| Characteristics | All | With acute kidney injury | | Without acute kidney injury | | *p* value |
| --- | --- | --- | --- | --- | --- | --- |
|  |  | n | median (IQR) | n | median (IQR) |  |
| *Complete blood counts* |  |  |  |  |  |  |
| Hemoglobin (g/dL) | 257 | 139 | 13.3 (10.6−15.5) | 118 | 13.0 (12.1−14.3) | 0.788 |
| Hematocrit (%) | 257 | 139 | 38.8 (31.2−45.8) | 118 | 40.0 (36.3−43.2) | 0.305 |
| WBC (×10^3^ cells/µL) | 257 | 139 | 20.4 (13.1−34.7) | 118 | 11.0 (8.3−15.7) | <0.001 |
| Neutrophils (%) | 257 | 139 | 80.5 (75.7−85.9) | 118 | 74.6 (59.2−82.7) | <0.001 |
| Lymphocytes (%) | 257 | 139 | 10.5 (7.2−14.0) | 118 | 15.2 (9.4−24.4) | <0.001 |
| PLT (×10^3^ cells/µL) | 257 | 139 | 70.0 (34.0−142.0) | 118 | 226.0 (182.2−270.0) | <0.001 |
| PT (sec) | 254 | 136 | 17.4 (15.6−21.5) | 118 | 16.4 (15.1−18.6) | 0.036 |
| INR | 258 | 140 | 1.4 (1.2−1.8) | 118 | 1.3 (1.1−1.5) | 0.019 |
| aPTT (sec) | 253 | 136 | 39.4 (30.8−50.5) | 117 | 35.2 (26.7-44.9) | 0.005 |
| Fibrinogen (mg/dL) | 252 | 134 | 455.5 (235.2−971.0) | 118 | 312.5 (215.8−695.0) | 0.013 |
| D-dimer (μg/mL) | 254 | 136 | 2.5 (1.1−6.5) | 118 | 1.0 (0.5−3.0) | <0.001 |
| Incoagulable blood on 20WBCT^*^, n (%) |  |  |  |  |  |  |
| Yes | 149 | 114 | 76.5 | 35 | 23.5 | <0.001 |
| No | 109 | 26 | 23.9 | 83 | 76.1 |  |
| *Blood chemistries* |  |  |  |  |  |  |
| Blood sugar (mg/dL) | 258 | 140 | 128.5 (112.0−150.8) | 118 | 120.0 (101.0−136.0) | 0.002 |
| Serum urea (mg/dL) | 258 | 140 | 59.7 (29.1−145.5 | 118 | 24.0 (17.2−31.8) | <0.001 |
| Serum creatinine (mg/dL) | 258 | 140 | 2.2 (1.2−4.1) | 118 | 0.9 (0.7−1.0) | <0.001 |
| Serum urea/creatinine ratio | 258 | 140 | 31.9 (15.5−47.8) | 118 | 28.7 (20.2−39.5) | 0.532 |
| Sodium (mmol/L) | 258 | 140 | 132.6 (127.6−137.0) | 118 | 138.0 (135.2−140.0) | <0.001 |
| Potassium (mmol/L) | 258 | 140 | 4.3 (3.7−5.0) | 118 | 3.9 (3.5−4.2) | <0.001 |
| Chloride (mmol/L) | 258 | 140 | 100.2 (92.8−105.1) | 118 | 104.0 (101.0−106.7) | <0.001 |
| Bicarbonate (mmol/L) | 258 | 140 | 18.5 (16.0−21.3) | 118 | 22.0 (20.0−25.0) | <0.001 |
| Calcium (mg/dL) | 256 | 138 | 8.0 (7.2−8.6) | 118 | 8.7 (8.2−9.1) | <0.001 |
| Phosphate (mg/dL) | 256 | 138 | 5.4 (4.0−7.6) | 118 | 3.6 (2.9−4.5) | <0.001 |
| Creatine kinase (IU/L) | 256 | 138 | 940.3 (239.2−3602.6) | 118 | 117.4 (57.5−286.3) | <0.001 |
| Albumin (g/dL) | 256 | 138 | 3.1 (2.6−3.5) | 118 | 3.9 (3.5−4.2) | <0.001 |
| AST (IU/L) | 256 | 138 | 50.6 (26.4−114.0) | 118 | 21.6 (15.4−31.5) | <0.001 |
| ALT (IU/L) | 256 | 138 | 12.8 (7.0−27.0) | 118 | 7.8 (4.8−11.7) | <0.001 |
| *Urinalysis* |  |  |  |  |  |  |
| Specific gravity | 255 | 137 | 1.020 (1.015−1.020) | 118 | 1.020 (1.015−1.025) | 0.760 |
| Microscopic hematuria^*^, n (%) |  |  |  |  |  |  |
| Yes | 81 | 70 | 86.4 | 11 | 13.6 | <0.001 |
| No | 174 | 67 | 38.5 | 107 | 61.5 |  |
| Leukocyturia^*^, n (%) |  |  |  |  |  |  |
| Yes | 22 | 16 | 72.7 | 6 | 27.3 | 0.100 |
| No | 233 | 121 | 51.9 | 112 | 48.1 |  |
| Urine protein-to-creatinine ratio | 186 | 127 | 5.7 (1.3−19.4) | 59 | 0.1 (0.0−0.6) | <0.001 |
| Fe sodium (%) | 258 | 140 | 3.1 (1.1−7.5) | 118 | 0.9 (0.4−1.8) | <0.001 |
| Fe urea (%) | 250 | 135 | 28.5 (12.3−61.3) | 115 | 42.3 (29.9−64.9) | 0.001 |
| *Categorical data* |  |  |  |  |  |  |
| WBC (cells/µL)^*^, n (%) |  |  |  |  |  |  |
| >10 ×10^3^ | 194 | 126 | 64.9 | 68 | 35.1 | <0.001 |
| ≤10 ×10^3^ | 64 | 14 | 21.9 | 50 | 78.1 |  |
| Overt DIC^*^, n (%) |  |  |  |  |  |  |
| Yes | 147 | 103 | 70.1 | 44 | 29.9 | <0.001 |
| No | 111 | 37 | 33.3 | 74 | 66.7 |  |
| Capillary leakage^*^, n (%) |  |  |  |  |  |  |
| Yes | 35 | 30 | 85.7 | 5 | 14.3 | <0.001 |
| No | 223 | 110 | 49.3 | 113 | 50.7 |  |
| Creatine kinase (IU/L)^*^, n (%) |  |  |  |  |  |  |
| >500 | 104 | 87 | 83.7 | 17 | 16.3 | <0.001 |
| ≤500 | 152 | 51 | 33.6 | 101 | 66.4 |  |
| Blood sugar (mg/dL)^*^, n (%) |  |  |  |  |  |  |
| ≥150 | 53 | 37 | 69.8 | 16 | 30.2 | 0.017 |
| <150 | 205 | 103 | 50.2 | 102 | 49.8 |  |
| Serum sodium (mmol/L)^*^, n (%) |  |  |  |  |  |  |
| <135 mmol/L | 115 | 91 | 79.1 | 24 | 20.9 | <0.001 |
| ≥135 mmol/L | 143 | 49 | 34.3 | 94 | 65.7 |  |

Data are presented as median (interquartile range) unless otherwise noted. ^*^Data are presented as n (%). ALT, alanine aminotransferase; aPTT, activated partial thromboplastin time; AST, aspartate aminotransferase; DIC, disseminated intravascular coagulation; Fe, fractional excretion; INR, international normalized ration; IQR, interquartile range; PLT, platelet; PT, prothrombin time; WBC, white blood cell; 20WBCT, 20-minute whole blood clotting test.

**Table S6.** Management and outcomes among 258 adults with snakebite envenomation, Yangon, Myanmar, 2015−2016

| Characteristics | All | With acute kidney injury, n (%) | Without acute kidney injury, n (%) | *p* value |
| --- | --- | --- | --- | --- |
| *Management* |  |  |  |  |
| Time from bite to hospital (hours) |  |  |  |  |
| ≥1 | 198 | 115 (58.1) | 83 (41.9) | 0.037 |
| <1 | 60 | 25 (41.7) | 35 (58.3) |  |
| Time from bite to receiving antivenom (hours) |  |  |  |  |
| ≥2 | 174 | 107 (61.5) | 67 (38.5) | 0.007 |
| <2 | 76 | 32 (42.1) | 44 (57.9) |  |
| Type of antivenom |  |  |  |  |
| Monovalent | 161 | 86 (53.4) | 75 (46.6) | 0.423 |
| Polyvalent | 89 | 53 (59.6) | 36 (40.4) |  |
| Total dose of antivenom (mL), median (IQR) | 250 | 160.0 (100.0−200.0) | 80.0 (60.0−120.0) | <0.001 |
| Antibiotics at admission |  |  |  |  |
| Yes | 241 | 140 (58.1) | 101 (41.9) | <0.001 |
| No | 17 | 0 | 17 (100) |  |
| Site of management |  |  |  |  |
| Ward | 211 | 129 (61.1) | 82 (38.9) | <0.001 |
| ICU | 47 | 11 (23.4) | 36 (76.6) |  |
| Inotropic drugs |  |  |  |  |
| Yes | 46 | 43 (93.5) | 3 (6.5) | <0.001 |
| No | 212 | 97 (45.8) | 115 (54.2) |  |
| Hospital acquired infection |  |  |  |  |
| Yes | 32 | 23 (71.9) | 9 (28.1) | 0.052 |
| No | 226 | 117 (51.8) | 109 (48.2) |  |
| Panhypopituitarism |  |  |  |  |
| Yes | 15 | 14 (93.3) | 1 (6.7) | 0.004 |
| No | 243 | 126 (51.9) | 117 (48.1) |  |
| *Outcomes* |  |  |  |  |
| Hospitalization (days) , median (IQR) | 258 | 11.5 (6.0−17.8) | 3.0 (2.0−4.0) | <0.001 |
| Renal replacement therapy |  |  |  |  |
| Yes | 69 | 69 (100.0) | 0 (0) | <0.001 |
| No | 189 | 71 (37.6) | 118 (62.4) |  |
| Survival status |  |  |  |  |
| Deceased | 28 | 27 (96.4) | 1 (3.6) | <0.001 |
| Survivor | 230 | 113 (49.1) | 117 (50.9) |  |

ICU, intensive care unit; IQR, interquartile range.

**Table S7.** Clinical parameters among 164 adults bitten by *Viperidae* or presenting the clinical syndrome of *Viperidae* (86 patients with acute kidney injury at presentation and 78 patients without acute kidney injury)

| Characteristics | All | With acute kidney injury, n (%) | Without acute kidney injury, n (%) | *p* value |
| --- | --- | --- | --- | --- |
| *Local symptoms and signs* |  |  |  |  |
| Tender lymphadenitis |  |  |  |  |
| Yes | 123 | 77 (62.6) | 46 (37.4) | <0.001 |
| No | 41 | 9 (22.0) | 32 (78.0) |  |
| Swelling |  |  |  |  |
| Grades II-IV | 117 | 72 (61.5) | 45 (38.5) | <0.001 |
| Grades 0-I | 47 | 14 (29.8) | 33 (70.2) |  |
| *Non-specific symptoms and signs* |  |  |  |  |
| Vomiting |  |  |  |  |
| Yes | 87 | 63 (72.4) | 24 (27.6) | <0.001 |
| No | 77 | 23 (29.9) | 54 (70.1) |  |
| Abdominal pain |  |  |  |  |
| Yes | 41 | 32 (78.0) | 9 (22.0) | <0.001 |
| No | 123 | 54 (43.9) | 69 (56.1) |  |
| *Cardiovascular presentations* |  |  |  |  |
| Abnormal ECG |  |  |  |  |
| Yes | 54 | 40 (74.1) | 14 (25.9) | <0.001 |
| No | 110 | 46 (41.8) | 64 (58.2) |  |
| Hypotension |  |  |  |  |
| Yes | 46 | 39 (84.8) | 7 (15.2) | <0.001 |
| No | 118 | 47 (39.8) | 71 (60.2) |  |
| Shock |  |  |  |  |
| Yes | 41 | 38 (92.7) | 3 (7.3) | <0.001 |
| No | 123 | 48 (39.0) | 75 (61.0) |  |
| Conjunctival edema |  |  |  |  |
| Yes | 24 | 23 (95.8) | 1 (4.2) | <0.001 |
| No | 140 | 63 (45.0) | 77 (55.0) |  |
| Pulmonary edema |  |  |  |  |
| Yes | 13 | 13 (100.0) | 0 (0) | <0.001 |
| No | 151 | 73 (48.3) | 78 (51.7) |  |
| *Hemostatic presentations* |  |  |  |  |
| Systemic bleeding |  |  |  |  |
| Yes | 65 | 57 (87.7) | 8 (12.3) | <0.001 |
| No | 99 | 29 (29.3) | 70 (70.7) |  |
| Local bleeding |  |  |  |  |
| Yes | 17 | 12 (70.6) | 5 (29.4) | 0.185 |
| No | 147 | 74 (50.3) | 73 (49.7) |  |
| *Renal presentations* |  |  |  |  |
| Renal tenderness |  |  |  |  |
| Yes | 81 | 66 (81.5) | 15 (18.5) | <0.001 |
| No | 83 | 20 (24.1) | 63 (75.9) |  |

ECG, electrocardiography.

**Table S8.** Laboratory parameters among 164 adults bitten by *Viperidae* or presenting the clinical syndrome of *Viperidae* (86 patients with acute kidney injury at presentation and 78 patients without acute kidney injury)

| Characteristics | All | With acute kidney injury | | Without acute kidney injury | | *p* value |
| --- | --- | --- | --- | --- | --- | --- |
|  |  | n | median (IQR) | n | median (IQR) |  |
| *Complete blood counts* |  |  |  |  |  |  |
| Hemoglobin (g/dL) | 163 | 85 | 12.7 (10.2−15.6) | 78 | 12.9 (12.1−14.3) | 0.371 |
| Hematocrit (%) | 163 | 85 | 37.8 (29.2−46.1) | 78 | 38.6 (35.5−42.4) | 0.232 |
| WBC (×10^3^ cells/µL) | 163 | 85 | 20.9 (12.4−36.9) | 78 | 11.4 (8.4−15.7) | <0.001 |
| Neutrophils (%) | 163 | 85 | 79.4 (73.7−85.2) | 78 | 73.4 (58.8−82.7) | 0.003 |
| Lymphocytes (%) | 163 | 85 | 11.5 (7.6−14.6) | 78 | 16.0 (9.4−25.1) | <0.001 |
| PLT (×10^3^ cells/µL) | 163 | 85 | 57.0 (31.5−115.0) | 78 | 220.0 (178.0−270.0) | <0.001 |
| PT (sec) | 162 | 84 | 17.0 (15.5−20.7) | 78 | 16.4 (15.1−18.7) | 0.286 |
| INR | 164 | 86 | 1.3 (1.2−1.7) | 78 | 1.3 (1.1−1.5) | 0.244 |
| aPTT (sec) | 161 | 84 | 38.8 (30.0−49.7) | 77 | 35.2 (27.0−44.9) | 0.039 |
| Fibrinogen (mg/dL) | 161 | 83 | 509.0 (281.9−940.0) | 78 | 286.0 (170.8−574.5) | <0.001 |
| D-dimer (μg/mL) | 162 | 84 | 2.5 (0.9−6.5) | 78 | 1.6 (0.5−5.1) | 0.024 |
| Incoagulable blood on 20WBCT*, n (%) |  |  |  |  |  |  |
| Yes | 117 | 82 | 70.1 | 35 | 29.9 | <0.001 |
| No | 47 | 4 | 8.5 | 43 | 91.5 |  |
| *Blood chemistries* |  |  |  |  |  |  |
| Blood sugar (mg/dL) | 164 | 86 | 130.0 (113.8−164.8) | 78 | 120.5 (101.8−139.0) | 0.003 |
| Serum urea (mg/dL) | 164 | 86 | 70.8 (31.1−152.0) | 78 | 24.6 (17.8−31.8) | <0.001 |
| Serum creatinine (mg/dL) | 164 | 86 | 3.5 (2.5−5.9) | 78 | 0.9 (0.7−1.0) | <0.001 |
| Sodium (mmol/L) | 164 | 86 | 131.2 (127.6−136.0) | 78 | 138.0 (135.4−140.0) | <0.001 |
| Potassium (mmol/L) | 164 | 86 | 4.4 (3.9−5.1) | 78 | 3.9 (3.5−4.4) | <0.001 |
| Chloride (mmol/L) | 164 | 86 | 99.8 (92.4−104.0) | 78 | 104.0 (101.2−106.1) | <0.001 |
| Bicarbonate (mmol/L) | 164 | 86 | 18.4 (15.6−20.9) | 78 | 21.7 (19.4−25.0) | <0.001 |
| Calcium (mg/dL) | 164 | 86 | 7.9 (7.1−8.5) | 78 | 8.7 (8.2−9.1) | <0.001 |
| Phosphate (mg/dL) | 164 | 86 | 6.1 (4.5−7.6) | 78 | 3.5 (2.8−4.6) | <0.001 |
| Creatine kinase (IU/L) | 164 | 86 | 1290.4 (277.4−3751.9) | 78 | 129.1 (53.7−364.5) | <0.001 |
| Albumin (g/dL) | 164 | 86 | 3.1 (2.6−3.4) | 78 | 4.0 (3.6−4.3) | <0.001 |
| AST (IU/L) | 164 | 86 | 57.8 (29.4−138.6) | 78 | 21.2 (16.2−28.8) | <0.001 |
| ALT (IU/L) | 164 | 86 | 13.2 (7.0−30.8) | 78 | 7.6 (5.2−10.9) | <0.001 |
| *Urinalysis* |  |  |  |  |  |  |
| Specific gravity | 162 | 84 | 1.020 (1.015−1.020) | 78 | 1.020 (1.015−1.025) | 0.265 |
| Microscopic hematuria*, n (%) |  |  |  |  |  |  |
| Yes | 61 | 51 | 83.6 | 10 | 16.4 | <0.001 |
| No | 101 | 33 | 32.7 | 68 | 67.3 |  |
| Leukocyturia*, n (%) |  |  |  |  |  |  |
| Yes | 13 | 10 | 76.9 | 3 | 23.1 | 0.110 |
| No | 149 | 74 | 49.7 | 75 | 50.3 |  |
| Urine protein to creatinine ratio | 118 | 78 | 5.6 (1.2−22.8) | 40 | 0.2 (0.0−2.2) | <0.001 |
| Fe sodium (%) | 164 | 86 | 4.0 (1.9−14.7) | 78 | 0.9 (0.4−1.8) | <0.001 |
| Fe urea (%) | 159 | 83 | 34.7 (17.1−105.3) | 76 | 42.0 (30.2−63.8) | 0.249 |
| *Categorical data* |  |  |  |  |  |  |
| WBC (cells/µL)*, n (%) |  |  |  |  |  |  |
| >10 ×10^3^ | 122 | 75 | 61.5 | 47 | 38.5 | <0.001 |
| ≤10 ×10^3^ | 41 | 10 | 24.4 | 31 | 75.6 |  |
| Overt DIC*, n (%) |  |  |  |  |  |  |
| Yes | 100 | 64 | 64.0 | 36 | 36.0 | <0.001 |
| No | 64 | 22 | 34.4 | 42 | 65.6 |  |
| Capillary leakage*, n (%) |  |  |  |  |  |  |
| Yes | 19 | 17 | 89.5 | 2 | 10.5 | 0.001 |
| No | 145 | 69 | 47.6 | 76 | 52.4 |  |
| Creatine kinase (IU/L)*, n (%) |  |  |  |  |  |  |
| >500 | 69 | 57 | 82.6 | 12 | 17.4 | <0.001 |
| ≤500 | 95 | 29 | 30.5 | 66 | 69.5 |  |
| Blood sugar (mg/dL)^*^, n (%) |  |  |  |  |  |  |
| ≥150 | 38 | 27 | 71.1 | 11 | 28.9 | 0.015 |
| <150 | 126 | 59 | 46.8 | 67 | 53.2 |  |
| Serum sodium (mmol/L)*, n (%) |  |  |  |  |  |  |
| <135 mmol/L | 77 | 61 | 79.2 | 16 | 20.8 | <0.001 |
| ≥135 mmol/L | 87 | 25 | 28.7 | 62 | 71.3 |  |

Data are presented as median (interquartile range) unless otherwise noted. *Data are presented as n (%).AKI, acute kidney injury; ALT, alanine aminotransferase; aPTT, activated partial thromboplastin time; AST, aspartate aminotransferase; DIC, disseminated intravascular coagulation; Fe, fractional excretion; INR, international normalized ration; IQR, interquartile range; PLT, platelet; PT, prothrombin time; WBC, white blood cell; 20WBCT, 20-minute whole blood.

**Table S9.** Management and outcomes among 164 adults bitten by *Viperidae* or presenting the clinical syndrome of *Viperidae* (86 patients with acute kidney injury at presentation and 78 patients without acute kidney injury)

| Characteristics | All | With acute kidney injury, n (%) | Without acute kidney injury, n (%) | *p* value |
| --- | --- | --- | --- | --- |
| *Management* |  |  |  |  |
| Time from bite to hospital (hours) |  |  |  |  |
| ≥1 | 125 | 71 (56.8) | 54 (43.2) | 0.069 |
| <1 | 39 | 15 (38.5) | 24 (61.5) |  |
| Time from bite to receiving antivenom (hours) |  |  |  |  |
| ≥2 | 112 | 68 (60.7) | 44 (39.3) | 0.021 |
| <2 | 44 | 17 (38.6) | 27 (61.4) |  |
| Type of antivenom |  |  |  |  |
| Monovalent | 103 | 46 (44.7) | 57 (55.3) | 0.001 |
| Polyvalent | 53 | 39 (73.6) | 14 (26.4) |  |
| Total dose of antivenom (mL) |  |  |  |  |
| >160 | 43 | 31 (72.1) | 12 (27.9) | 0.011 |
| ≤160 | 113 | 54 (47.8) | 59 (52.2) |  |
| Antibiotics at admission |  |  |  |  |
| Yes | 158 | 86 (54.4) | 72 (45.6) | 0.010 |
| No | 6 | 0 (0) | 6 (100.0) |  |
| Inotropic drugs |  |  |  |  |
| Yes | 29 | 27 (93.1) | 2 (6.9) | <0.001 |
| No | 135 | 59 (43.7) | 76 (56.3) |  |
| Hospital acquired infection |  |  |  |  |
| Yes | 18 | 13 (72.2) | 5 (27.8) | 0.126 |
| No | 146 | 73 (50.0) | 73 (50.0) |  |
| Panhypopituitarism |  |  |  |  |
| Yes | 11 | 10 (90.9) | 1 (9.1) | 0.020 |
| No | 153 | 76 (49.7) | 77 (50.3) |  |
| *Outcomes* |  |  |  |  |
| Hospitalization (days), median (IQR) | 164 | 13.0 (7.8−19.2) | 3.0 (3.0−4.0) | <0.001 |
| Renal replacement therapy |  |  |  |  |
| Yes | 51 | 51 (100.0) | 0 (0) | <0.001 |
| No | 113 | 35 (31.0) | 78 (69.0) |  |
| Survival status |  |  |  |  |
| Deceased | 17 | 16 (94.1) | 1 (5.9) | 0.001 |
| Survivor | 147 | 70 (47.6) | 77 (52.4) |  |

AKI, acute kidney injury; IQR, interquartile range.

**Table S10.** Clinical parameters among 128 adults bitten by *Viperidae* or presenting the clinical syndrome of *Viperidae* (50 patients developed acute kidney injury during hospitalization and 78 patients without acute kidney injury)

| Characteristics | All | With acute kidney injury, n (%) | Without acute kidney injury, n (%) | *p* value |
| --- | --- | --- | --- | --- |
| *Local symptoms and signs* |  |  |  |  |
| Tender lymphadenitis |  |  |  |  |
| Yes | 88 | 42 (47.7) | 46 (52.3) | 0.005 |
| No | 40 | 8 (20.0) | 32 (80.0) |  |
| Swelling |  |  |  |  |
| Grades II-IV | 82 | 37 (45.1) | 45 (54.9) | 0.092 |
| Grades 0-I | 46 | 13 (28.3) | 33 (71.7) |  |
| *Non-specific symptoms and signs* |  |  |  |  |
| Vomiting |  |  |  |  |
| Yes | 59 | 35 (59.3) | 24 (40.7) | <0.001 |
| No | 69 | 15 (21.7) | 54 (78.3) |  |
| Abdominal pain |  |  |  |  |
| Yes | 25 | 16 (64.0) | 9 (36.0) | 0.009 |
| No | 103 | 34 (33.0) | 69 (67.0) |  |
| *Cardiovascular presentations* |  |  |  |  |
| Abnormal ECG |  |  |  |  |
| Yes | 35 | 21 (60.0) | 14 (40.0) | 0.006 |
| No | 93 | 29 (31.2) | 64 (68.8) |  |
| Hypotension |  |  |  |  |
| Yes | 25 | 18 (72.0) | 7 (28.0) | <0.001 |
| No | 103 | 32 (31.1) | 71 (68.9) |  |
| Shock |  |  |  |  |
| Yes | 17 | 14 (82.4) | 3 (17.6) | <0.001 |
| No | 111 | 36 (32.4) | 75 (67.6) |  |
| Conjunctival edema |  |  |  |  |
| Yes | 14 | 13 (92.9) | 1 (7.1) | <0.001 |
| No | 114 | 37 (32.5) | 77 (67.5) |  |
| Pulmonary edema |  |  |  |  |
| Yes | 7 | 7 (100.0) | 0 (0) | 0.001 |
| No | 121 | 43 (35.5) | 78 (64.5) |  |
| *Hemostatic presentations* |  |  |  |  |
| Systemic bleeding |  |  |  |  |
| Yes | 34 | 26 (76.5) | 8 (23.5) | <0.001 |
| No | 94 | 24 (25.5) | 70 (74.5) |  |
| Local bleeding |  |  |  |  |
| Yes | 8 | 3 (37.5) | 5 (62.5) | 1.000 |
| No | 120 | 47 (39.2) | 73 (60.8) |  |
| *Renal presentations* |  |  |  |  |
| Renal tenderness |  |  |  |  |
| Yes | 44 | 29 (65.9) | 15 (34.1) | <0.001 |
| No | 84 | 21 (25.0) | 63 (75.0) |  |

ECG, electrocardiography

**Table S11.** Laboratory parameters among 128 adults bitten by *Viperidae* or presenting the clinical syndrome of *Viperidae* (50 patients developed acute kidney injury during hospitalization and 78 patients without acute kidney injury)

| Characteristics | All | With acute kidney injury | | Without acute kidney injury | | *p* value |
| --- | --- | --- | --- | --- | --- | --- |
|  |  | n | median (IQR) | n | median (IQR) |  |
| *Complete blood counts* |  |  |  |  |  |  |
| Hemoglobin (g/dL) | 128 | 50 | 13.4 (11.7−15.6) | 78 | 12.9 (12.1−14.3) | 0.423 |
| Hematocrit (%) | 128 | 50 | 39.0 (34.0−45.3) | 78 | 38.6 (35.5−42.4) | 0.922 |
| WBC (×10^3^ cells/µL) | 128 | 50 | 17.8 (13.8−33.8) | 78 | 11.4 (8.4−15.7) | <0.001 |
| Neutrophils (%) | 128 | 50 | 82.9 (79.6−87.7) | 78 | 73.4 (58.8−82.7) | <0.001 |
| Lymphocytes (%) | 128 | 50 | 8.5 (6.6−12.6) | 78 | 16.0 (9.4−25.1) | <0.001 |
| PLT (×10^3^ cells/µL) | 128 | 50 | 124.0 (39.0−183.2) | 78 | 220.0 (178.0−270.0) | <0.001 |
| PT (sec) | 126 | 48 | 18.4 (15.8−21.5) | 78 | 16.4 (15.1−18.7) | 0.113 |
| INR | 128 | 50 | 1.5 (1.2−1.9) | 78 | 1.3 (1.1−1.5) | 0.051 |
| aPTT (sec) | 127 | 48 | 38.8 (30.7−52.2) | 77 | 35.2 (27.0−44.9) | 0.121 |
| Fibrinogen (mg/dL) | 125 | 47 | 392.0 (136.0−955.0) | 78 | 286.0 (170.8−574.5) | 0.304 |
| D-dimer (μg/mL) | 126 | 48 | 2.7 (1.2−7.6) | 78 | 1.6 (0.5−5.1) | 0.038 |
| Incoagulable blood on 20WBCT*, n (%) |  |  |  |  |  |  |
| Yes | 79 | 44 | 55.7 | 35 | 44.3 | <0.001 |
| No | 49 | 6 | 12.2 | 43 | 87.8 |  |
| *Blood chemistries* |  |  |  |  |  |  |
| Blood sugar (mg/dL) | 128 | 50 | 126.5 (107.8−146.2) | 78 | 120.5 (101.8−139.0) | 0.095 |
| Serum urea (mg/dL) | 128 | 50 | 49.5 (28.6−134.7) | 78 | 24.6 (17.8−31.8) | <0.001 |
| Serum creatinine (mg/dL) | 128 | 50 | 1.1 (0.8−1.2) | 78 | 0.9 (0.7−1.0) | 0.002 |
| Sodium (mmol/L) | 128 | 50 | 133.0 (127.0−138.0) | 78 | 138.0 (135.4−140.0) | <0.001 |
| Potassium (mmol/L) | 128 | 50 | 4.1 (3.4−4.6) | 78 | 3.9 (3.5−4.4) | 0.433 |
| Chloride (mmol/L) | 128 | 50 | 101.7 (92.2−107.8) | 78 | 104.0 (101.2−106.1) | 0.028 |
| Bicarbonate (mmol/L) | 128 | 50 | 19.4 (17.2−23.0) | 78 | 21.7 (19.4−25.0) | 0.002 |
| Calcium (mg/dL) | 126 | 48 | 8.3 (7.6−8.8) | 78 | 8.7 (8.2−9.1) | 0.011 |
| Phosphate (mg/dL) | 126 | 48 | 4.8 (3.0−7.0) | 78 | 3.5 (2.8−4.6) | 0.007 |
| Creatine kinase (IU/L) | 126 | 48 | 688.6 (145.4−2681.9) | 78 | 129.1 (53.7−364.5) | <0.001 |
| Albumin (g/dL) | 126 | 48 | 3.1 (2.6−3.7) | 78 | 4.0 (3.6−4.3) | <0.001 |
| AST (IU/L) | 126 | 48 | 41.0 (19.8−86.8) | 78 | 21.2 (16.2−28.8) | <0.001 |
| ALT (IU/L) | 126 | 48 | 11.4 (6.2−20.6) | 78 | 7.6 (5.2−10.9) | 0.001 |
| *Urinalysis* |  |  |  |  |  |  |
| Specific gravity | 127 | 49 | 1.020 (1.015−1.025) | 78 | 1.020 (1.015−1.025) | 0.904 |
| Microscopic hematuria*, n (%) |  |  |  |  |  |  |
| Yes | 27 | 17 | 63.0 | 10 | 37.0 | 0.007 |
| No | 100 | 32 | 32.0 | 68 | 68.0 |  |
| Leukocyturia*, n (%) |  |  |  |  |  |  |
| Yes | 9 | 6 | 66.7 | 3 | 33.3 | 0.087 |
| No | 118 | 43 | 36.4 | 75 | 63.6 |  |
| Urine protein to creatinine ratio | 84 | 44 | 6.1 (1.8−18.0) | 40 | 0.2 (0.0−2.2) | <0.001 |
| Fe sodium (%) | 128 | 50 | 1.2 (0.6−3.5) | 78 | 0.9 (0.4−1.8) | 0.158 |
| Fe urea (%) | 124 | 48 | 19.7 (6.7−40.2) | 76 | 42.0 (30.2−63.8) | <0.001 |
| *Categorical data* |  |  |  |  |  |  |
| WBC (cells/µL)*, n (%) |  |  |  |  |  |  |
| >10 ×10^3^ | 94 | 47 | 50.0 | 47 | 50.0 | <0.001 |
| ≤10 ×10^3^ | 34 | 3 | 8.8 | 31 | 91.2 |  |
| Overt DIC*, n (%) |  |  |  |  |  |  |
| Yes | 72 | 36 | 50.0 | 36 | 50.0 | 0.007 |
| No | 56 | 14 | 25.0 | 42 | 75.0 |  |
| Capillary leakage*, n (%) |  |  |  |  |  |  |
| Yes | 14 | 12 | 85.7 | 2 | 14.3 | <0.001 |
| No | 114 | 38 | 33.3 | 76 | 66.7 |  |
| Creatine kinase (IU/L)*, n (%) |  |  |  |  |  |  |
| >500 | 40 | 28 | 70.0 | 12 | 30.0 | <0.001 |
| ≤500 | 86 | 20 | 23.3 | 66 | 76.7 |  |
| Serum sodium (mmol/L)*, n (%) |  |  |  |  |  |  |
| <135 mmol/L | 45 | 29 | 64.4 | 16 | 35.6 | <0.001 |
| ≥135 mmol/L | 83 | 21 | 25.3 | 62 | 74.7 |  |

Data are presented as median (interquartile range) unless otherwise noted. *Data are presented as n (%).AKI, acute kidney injury; ALT, alanine aminotransferase; aPTT, activated partial thromboplastin time; AST, aspartate aminotransferase; DIC, disseminated intravascular coagulation; Fe, fractional excretion; IQR, interquartile range; INR, international normalized ration; PLT, platelet; PT, prothrombin time; WBC, white blood cell; 20WBCT, 20-minute whole blood.

**Table S12.** Management and outcomes among 128 adults bitten by *Viperidae* or presenting the clinical syndrome of *Viperidae* (50 patients developed acute kidney injury during hospitalization and 78 patients without acute kidney injury)

| Characteristics | All | With acute kidney injury, n (%) | Without acute kidney injury, n (%) | *p* value |
| --- | --- | --- | --- | --- |
| *Management* |  |  |  |  |
| Time from bite to hospital (hours) |  |  |  |  |
| ≥1 | 94 | 40 (42.6) | 54 (57.4) | 0.254 |
| <1 | 34 | 10 (29.4) | 24 (70.6) |  |
| Time from bite to receiving antivenom (hours) |  |  |  |  |
| ≥2 | 80 | 36 (45.0) | 44 (55.0) | 0.341 |
| <2 | 41 | 14 (34.1) | 27 (65.9) |  |
| Type of antivenom |  |  |  |  |
| Monovalent | 94 | 37 (39.4) | 57 (60.6) | 0.552 |
| Polyvalent | 27 | 13 (48.1) | 14 (51.9) |  |
| Total dose of antivenom (mL) |  |  |  |  |
| >160 | 31 | 19 (61.3) | 12 (38.7) | 0.016 |
| ≤160 | 90 | 31 (34.4) | 59 (65.6) |  |
| Antibiotics at admission |  |  |  |  |
| Yes | 122 | 50 (41.0) | 72 (59.0) | 0.081 |
| No | 6 | 0 (0) | 6 (100.0) |  |
| Mechanical ventilator |  |  |  |  |
| Yes | 1 | 1 (100.0) | 0 (0) | 0.391 |
| No | 127 | 49 (38.6) | 78 (61.4) |  |
| Inotropic drugs |  |  |  |  |
| Yes | 15 | 13 (86.7) | 2 (13.3) | <0.001 |
| No | 113 | 37 (32.7) | 76 (67.3) |  |
| Hospital acquired infection |  |  |  |  |
| Yes | 15 | 10 (66.7) | 5 (33.3) | 0.040 |
| No | 113 | 40 (35.4) | 73 (64.6) |  |
| Panhypopituitarism |  |  |  |  |
| Yes | 4 | 3 (75.0) | 1 (25.0) | 0.299 |
| No | 124 | 47 (37.9) | 77 (62.1) |  |
| *Outcomes* |  |  |  |  |
| Hospitalization (days), median (IQR) | 128 | 10.0 (4.0−14.0) | 3.0 (3.0−4.0) | <0.001 |
| Renal replacement therapy |  |  |  |  |
| Yes | 17 | 17 (100.0) | 0 (0) | <0.001 |
| No | 111 | 33 (29.7) | 78 (70.3) |  |
| Survival status |  |  |  |  |
| Deceased | 9 | 8 (88.9) | 1 (11.1) | 0.002 |
| Survivor | 119 | 42 (35.3) | 77 (64.7) |  |

IQR, interquartile range.
